# Consistent alterations in faecal microbiomes of patients with primary sclerosing cholangitis independent of associated colitis

**DOI:** 10.1111/apt.15375

**Published:** 2019-06-28

**Authors:** Malte Rühlemann, Timur Liwinski, Femke‐Anouska Heinsen, Corinna Bang, Roman Zenouzi, Martin Kummen, Louise Thingholm, Marie Tempel, Wolfgang Lieb, Tom Karlsen, Ansgar Lohse, Johannes Hov, Gerald Denk, Frank Lammert, Marcin Krawczyk, Christoph Schramm, Andre Franke

**Affiliations:** ^1^ Kiel Germany; ^2^ Hamburg Germany; ^3^ Oslo Norway; ^4^ Munich Germany; ^5^ Homburg Germany; ^6^ Warsaw Poland

## Abstract

**Background:**

Single‐centre studies reported alterations of faecal microbiota in patients with primary sclerosing cholangitis (PSC). As regional factors may affect microbial communities, it is unclear if a microbial signature of PSC exists across different geographical regions.

**Aim:**

To identify a robust microbial signature of PSC independent of geography and environmental influences.

**Methods:**

We included 388 individuals (median age, 47 years; range, 15‐78) from Germany and Norway in the study, 137 patients with PSC (n = 75 with colitis), 118 with ulcerative colitis (UC) and 133 healthy controls. Faecal microbiomes were analysed by 16S rRNA gene sequencing (V1‐V2). Differences in relative abundances of single taxa were subjected to a meta‐analysis.

**Results:**

In both cohorts, microbiota composition (beta‐diversity) differed between PSC patients and controls (*P* < 0.001). Random forests classification discriminated PSC patients from controls in both geographical cohorts with an average area under the curve of 0.88. Compared to healthy controls, many new cohort‐spanning alterations were identified in PSC, such as an increase of Proteobacteria and the bile‐tolerant genus *Parabacteroides*, which were detected independent from geographical region. Associated colitis only had minor effects on microbiota composition, suggesting that PSC itself drives the faecal microbiota changes observed.

**Conclusion:**

Compared to healthy controls, numerous microbiota alterations are reproducible in PSC patients across geographical regions, clearly pointing towards a microbiota composition that is shaped by the disease itself and not by environmental factors. These reproducibly altered microbial populations might provide future insights into the pathophysiology of PSC.

## 1 INTRODUCTION

Primary sclerosing cholangitis (PSC) is a chronic progressive disease of the biliary system.^1^ There is no medical treatment available and patients have an increased risk of both hepatobiliary and bowel malignancies.^2^, ^3^ As a result, most patients decease or receive liver transplantation 15‐20 years after diagnosis.^4^ The pathogenesis is still unclear, although a dysregulated immune reaction at the epithelial barrier in the intestine and the biliary system may play a key role. Co‐occurrence of inflammatory bowel disease (IBD), typically in form of a mild pancolitis, has been reported in up to 80% of patients with PSC, and at least 2%‐7.5% of IBD‐patients develop PSC.^1^, ^5^, ^6^, ^7^ It has been postulated that PSC‐associated colitis (PSC‐IBD) represents a distinct IBD entity alongside Crohn's disease and UC.^8^, ^9^

Several genetic risk factors have been identified that are associated with PSC, but collectively they only contribute to a small fraction of disease susceptibility.^1^ Given the minor role of genetic variation, environmental factors likely play a major role in the pathogenesis of PSC. Of these, the microbiome has emerged as one of the most important potential environmental players in chronic inflammatory diseases. Gut microbiome alterations have been identified in several metabolic and inflammatory diseases,^10^, ^11^, ^12^ and recent reports have demonstrated alterations in both the faecal and mucosal microbiota in patients with PSC.^13^, ^14^ The faecal microbiota of PSC patients has been characterised by a distinct profile compared to healthy controls and UC patients, including an overabundance of *Veillonella* in a Norwegian and a Czech cohort.^13^, ^15^ However, a classification between PSC and UC in a Norwegian cohort based on the abundance of *Veillonella* could not be validated in a German population.^16^ In a Belgian cohort, stool samples of PSC patients showed an increased abundance of the genera *Fusobacterium, Enterococcus*,* Lactobacillus* and *Streptococcus* compared to healthy individuals.^17^

In summary, it is unclear whether the microbial signature so far described in PSC single‐centre cohorts is centre‐specific or if a PSC‐specific microbial signature across different geographical regions exists. Cross‐regional analysis of faecal microbiota of patients with PSC might reveal general patterns of microbial perturbation, which could elucidate the role of the microbiome in PSC and PSC‐IBD and provide the basis for a better understanding of their possible pathogenetic significance in further mechanistic and clinical longitudinal studies.

## 2 PATIENTS AND METHODS

Seventy‐four nontransplanted German patients with PSC (n = 37 PSC only, n = 37 PSC‐IBD) were recruited at the University Medical Center Hamburg‐Eppendorf. In addition, a German UC study cohort (n = 88) and 95 German healthy individuals (controls) were recruited for comparison by the PopGen Biobank.^18^

Furthermore, 63 nontransplanted Norwegian patients with PSC (n = 25 PSC only, n = 38 PSC‐IBD), 30 UC patients without PSC and 38 controls were recruited at the Norwegian PSC Research Center Biobank at Oslo University Hospital Rikshospitalet.^13^

PSC was diagnosed based on cholangiography and liver biopsy (if required) according to most recent guidelines.^19^, ^20^ All individuals underwent extensive screening for potential confounding (see Methods S1 for exclusion criteria)**.** The characteristics of both study populations are summarised in Table 1 **.**

**Table 1:** Demographic and clinical characteristics of the German and Norwegian study populations

German	Controls	PSC only	PSC‐IBD	UC
Total number	n = 95	n = 37	n = 37	n = 88
General information				
Age, median years (min‐max)	47 (19‐64)	51 (18‐73)*	46.5 (15‐73)	45 (19‐78)
Gender (female)	51.6% (n = 49)	32.4% (n = 12)	43.2% (n = 16)	61.4% (n = 54)
BMI, median kg/m^2^ (min‐max)	22.8 (20.2‐24.9)	23.7 (17.9‐32)**	23.6 (15.8‐34.3)*	24.8 (17.0‐36.5)***
Smoking (yes)	16.8% (n = 16)	8.1% (n = 3)	0%**	3.4% (n = 3)***
Dietary data				
Available	89.5% (n = 85)	83.8% (n = 31)	76.7% (n = 28)	86.4% (n = 76)
Daily intake, median (min‐max)				
Energy (kJ)	9025 (4,313‐23,006)	9961 (5,150‐18,249)	10 153 (4,218‐19,630)	9304 (5040‐19 609)
Carbohydrates (g)	215.9 (93.8‐772.9)	239.6 (124.7‐511.0)	275.3 (91.8‐588.1)	242.8 (103.3‐483.9)
Fibre (g)	20.1 (9.9‐25.5)	21.3 (12.9‐34.1)	24.1 (10.6‐46.5)	21.7 (10.4‐40.7)
Fat (g)	98.0 (46.4‐223.2)	105.2 (49.3‐202.3)	108.2 (45.1‐191.0)	95.8 (43.6‐203.3)
Protein (g)	77.3 (32.4‐206.0)	90.7 (45.0‐182.5)	84.4 (39.7‐136.1)	78.8 (41.4‐156.7)
Water (L)	3.15 (1.05‐7.73)	2.67 (1.47‐7.15)*	2.83 (1.43‐4.41)	2.79 (1.09‐7.32)
Faecal Calprotectin (fCAL)				
Median (µg/g) (Q1‐Q3)	27.3 (15.6‐40.9)	20 (10‐52.4)	29.4 (10‐110)	43.3 (18.3‐190.8)
fCAL low (<50 µg/g), %	80% (n = 42)	73.0% (n = 27)	56.8 (n = 21)	52.3% (n = 46)
fCAL elevated (50‐200 µg/g), %	19.2% (n = 10)	13.5% (n = 5)	27.0% (n = 10)	22.7% (n = 20)
fCAL high (>200 µg/g) %	0	13.5 (n = 5)	16.2 (n = 6)	25 (n = 22)
NA	n = 43	—		—
PSC additional information				
Years since PSC diagnosis, median (min‐max)	—	6.5 (0‐35)	9.0 (1‐28)	—
Cirrhosis (yes)	—	5.4% (n = 2)	5.4% (n = 2)	—
ALT, median U/L (min‐max)	—	37 (11‐165, NA = 10)	38.5 (13‐286, NA = 15)	—
AP, median U/L (min‐max)	—	116 (61‐590, NA = 10)	125 (44‐332, NA = 15)	—
Bilirubin, median U/L (min‐max)	—	10.3 (5.1‐35.9, NA = 11)	11.97 (3.4‐34.2, NA = 16)	—
Medication (%)				
UDCA	—	97.3 (n = 36)	94.6 (n = 35)	—
5‐ASA	—	2.7 (n = 1)	83.8 (n = 31)	79.5 (n = 80)
Azathioprine	—	5.4 (n = 2)	13.5 (n = 5)	30.7 (n = 27)
Budesonide	—	—	5.4 (n = 2)	31.8 (n = 28)
Biologics (Adalimumab, Infliximab)	—	—	5.4 (n = 2)	15.9 (n = 14)
PPI	—	—	—	—
Statins	—	—	—	—
Norwegian	Controls	PSC only	PSC‐IBD	UC
Total number	n = 38	n = 25	n = 38	n = 30
General information				
Age, median years (min‐max)	47 (35‐61)	46 (31‐66)	48 (21‐69)	42.5 (25‐69)
Gender (female) (%)	36.8 (n = 14)	36 (n = 9)	31.6 (n = 12)	53.3 (n = 16)
BMI, median kg/m^2^ (min‐max)	26 (19.4‐39.4)	26.0 (17.8‐32.2)	24.0 (17.7‐34.7)	24.5 (21.4‐34.3)
Smoking (yes) (%)	15.8 (n = 6)	0	2.6 (n = 1)	0*
PSC additional information				
Years since PSC diagnosis, median (min‐max)	—	7.8 (2.1‐31.7)	9.6 (1.4‐28.8)	—
Signs of impaired liver function (yes) (%)	—	4 (n = 1)	2.6 (n = 1)	—
ALT, median U/L (min‐max)	—	65.5 (16‐258), NA = 3)	54 (14‐331), NA = 2)	—
AP, median U/L (min‐max)	—	192 (50‐548, NA = 4)	130 (30‐589, NA = 2)	—
Bilirubin, median U/L (min‐max)	—	13.5 (6‐114, NA = 3)	13 (6‐44 NA = 3)	—
Medication (%)				
UDCA	—	36 (n = 9)	26.3 (n = 10)	—
5‐ASA	—	4 (n = 1)	57.9 (n = 22)	76.7 (n = 23)
Azathioprine	—	4 (n = 1)	15.8 (n = 6)	23.3 (n = 7)
Budesonide	—	4 (n = 1)	2.6 (n = 1)	6.7 (n = 2)
Biologics (Adalimumab, Infliximab)	—	—	2.6 (n = 1)	40 (n = 12)
PPI	—	—	2.6 (n = 1)	6.7 (n = 2)
Statins	—	16 (n = 4)	5.2 (n = 2)	—

Only medication taken by at least two patients is listed.

ALT, alanine aminotransferase; AP, alkaline phosphatase; ASA5, 5‐aminosalicylic acid; BMI, body mass index; PPI, proton pump inhibitors; PSC, primary sclerosing cholangitis; Q1, first quartile; Q3, third quartile; UC, ulcerative colitis; NA, not available.

* *P* < 0.05; **P < 0.01; ***P < 0.001.

The study was approved by the local ethics committees in Hamburg and Kiel (A148/14 and MC‐111/15) and the Regional Committee for Medical and Health Research Ethics in South‐Eastern Norway (reference 2012/286b). All participants gave their written informed consent.

### 2.1 Assessment of dietary patterns

Dietary data were collected for 220 individuals (Table 1) of the German study cohort using standardised and validated food frequency questionnaires of the German Institute of Human Nutrition. Translation into nutrients was performed via the German Food Code and Nutrient Database (vII.3).^21^

### 2.2 Stool sample processing and sequencing

Samples were collected and subjected to DNA extraction as previously described for the respective cohorts.^13^, ^22^ The amplification and library preparation of the V1‐V2 region of the 16S rRNA gene using dual‐indexing was performed in a single facility (Methods S1). We chose V1‐V2 as target amplicon, as 2 300 bp sequencing covers the amplicon of 300‐320 bp almost entirely twice, thus assuring high quality data readout. Sequencing data were subjected to quality control and data processing to obtain count‐based relative abundance tables for operational taxonomic units (OTUs) and taxonomic levels from phylum to genus (Methods S1).

### 2.3 Data analysis

Data analyses were performed with R statistical programming language (v3.4.3).^23^

Differences in dietary patterns were evaluated using the log‐transformed average intake of the primary macronutrients protein, fat and carbohydrates (g/day), as well as fibre, water (both g/day), and total energy intake (kJ/day). To assess dietary differences of diseased individuals from healthy controls, permutational analysis of variance (PERMANOVA) was performed on Euclidean distances using residuals of dietary data after regression against sex, as sex is a known major predictor of dietary behaviour.

Shannon index, as a measure of within‐sample diversity (alpha‐diversity), and PERMANOVA on Bray‐Curtis dissimilarity, as a measure for beta‐diversity, were applied to investigate differences according to disease state and other co‐variables (Methods S1).

All abundances are based on a normalised number of counts, thus being relative abundances, this is always the case when the term ‘abundance’ is used throughout the text. To assess differential taxa abundances, we tested microbes that were identified as differentially abundant in previous studies in a first step (Methods S1), followed by a second step where we aimed to discover new associations. Details on the applied regression models and meta‐analysis are provided in the Methods S1. Taxa with significant signals in both respective geographical cohorts and the meta‐analysis were re‐analysed with inclusion of dietary co‐variables in the German cohort, for which food frequency questionnaire data was available, to correct for dietary effects.

Predictive performance of the identified taxonomic signature was evaluated using random forests classification^24^ (Methods S1). To evaluate model performance, receiver operating characteristic analysis was used. Additionally, F1 score and Matthews Correlation Coefficient (MCC) as weighted measures of true and false positives rates were calculated (Methods S1).

## 3 RESULTS

In total, we applied 16S rRNA gene sequencing to 257 German (n = 95 controls, n = 37 PSC only, n = 37 PSC‐IBD and n = 88 UC) and 131 Norwegian samples (n = 38 controls, n = 25 PSC only, n = 38 PSC‐IBD and n = 30 UC). We applied the same amplification and library preparation standard operating procedure within a single facility to all samples. For 220 of the 257 (85.6%) German study participants dietary data, assessed by standardised food frequency questionnaires, was available (Table 1).

### 3.1 Healthy Norwegian and German subjects share similar core microbiota

To determine the baseline similarities and differences between the faecal microbiota of German and Norwegian healthy volunteers, we compared the healthy controls of both cohorts. We found large proportions of the core microbiota (122 of 144 taxa, 84.7%) shared by healthy controls from both populations (Figures S1‐S3). The healthy Norwegian cohort displayed a significantly lower intra‐individual (alpha) diversity compared to the healthy German population (*P* = 0.006). We observed slight but significant differences in between‐sample diversity (beta‐diversity) between German and Norwegian controls (*P* = 0.01; *R* ^2^ = 0.021).

### 3.2 The faecal microbiota of patients with PSC is significantly different from both healthy controls and patients with UC

In both cohorts, between‐sample diversity (beta‐diversity) was significantly different between patients with PSC and controls (*P* < 0.001, respectively; *R* ^2^_GER_ = 0.028; *R* ^2^_NOR_ = 0.042). Differences in beta‐diversity between patients with PSC and UC were also significant but less pronounced in both cohorts (*P*_NOR_ = 0.016, R^2^_NOR_ = 0.027; *P*_GER_ = 0.013, *R* ^2^_GER_ = 0.015).

**Figure 1:**
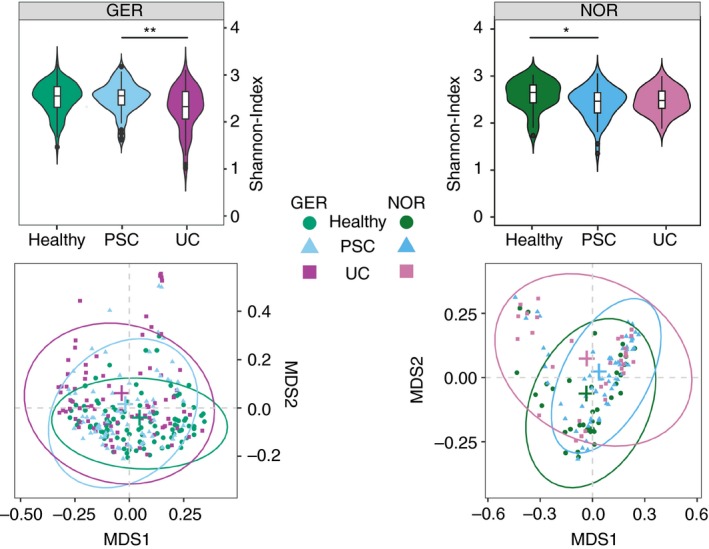
Violin plots of Shannon‐Index and the unconstrained ordination plots of the Bray‐Curtis dissimilarities for the German (GER) and the Norwegian (NOR) cohort. Ordination was performed on genus‐level abundances subsequently plotted for each cohort separately. Centroids of ellipses are marked by crosses in the respective colours. *P < 0.05; **P < 0.01

In the Norwegian cohort, mean within‐sample diversity (alpha‐diversity) of patients with PSC was reduced compared to controls (*P* = 0.001) and comparable to patients with UC (*P* > 0.05). In the German cohort however, alpha‐diversity of patients with PSC was comparable to controls (*P* > 0.05) and significantly increased in contrast with patients with UC (*P* = 0.01) (Figure 1).

### 3.3 Targeted analysis of taxa with previously reported association with PSC

A total of nine genera that were previously identified as differentially abundant^13^, ^17^ were analysed in a targeted approach aiming to reproduce the taxonomic signals. A detailed summary is provided in the Tables S1 and S2.

In both cohorts, an increased relative abundance in patients with PSC was displayed by *Veillonella* and *Streptococcus* (both *P*_META_ < 0.0001). In addition, an increased prevalence in patients with PSC was confirmed for *Lactobacillus* and *Enterococcus* (both *P*_META_ < 0.0001, respectively). Other taxa either showed inconsistent distribution patterns between cohorts or could not be recovered (with sufficient prevalence) in our samples.

### 3.4 Extensive microbiota alterations in patients with PSC

**Figure 2:**
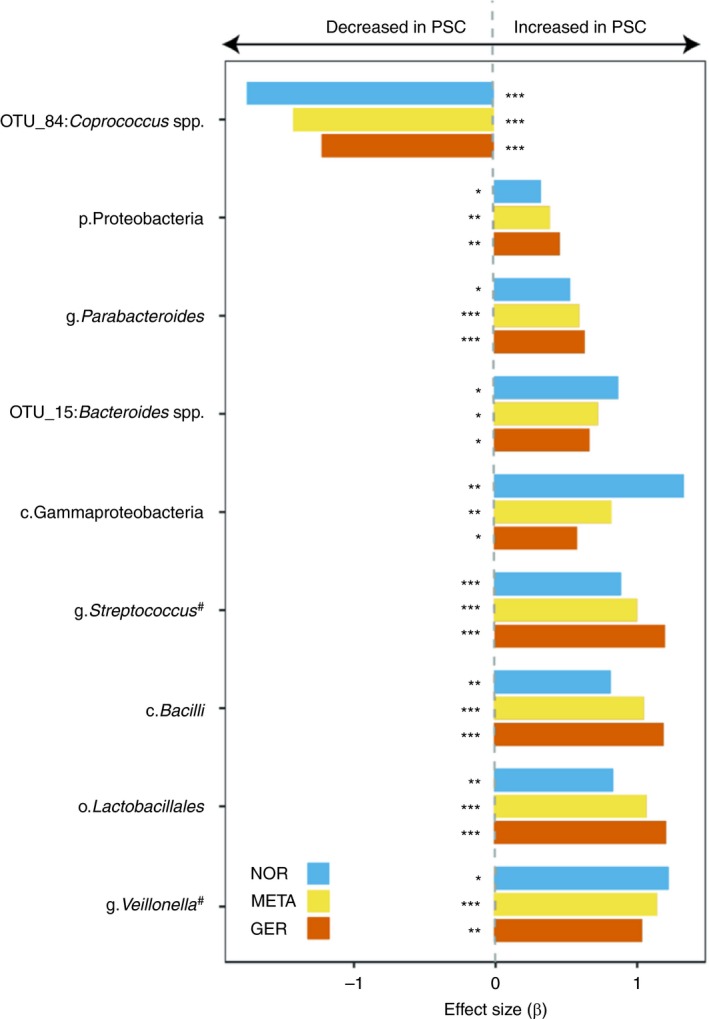
Significant and between‐cohort consistent results of differentially abundant taxa in PSC patients and controls. Only taxa with *P* < 0.05 in each cohort, *Q*_META_ < 0.05 and concordant directionality are shown. Taxa from Kummen *et al* or Sabino *et al* that could be replicated in both cohorts are marked with a pound (#) symbol. Base‐colours depict the respective cohort (blue: Germany; red: Norwegian) and the combined meta‐analysis result (yellow). Beta‐values larger than zero represent a higher abundance in PSC patients, taxa with beta‐values less than zero are less abundant in PSC patients. Details on the model coefficients and the resulting P‐values in the cohorts and the meta‐analysis can be found in S1‐S3. *P < 0.05; **P < 0.01; ***P < 0.001

**Figure 3:**
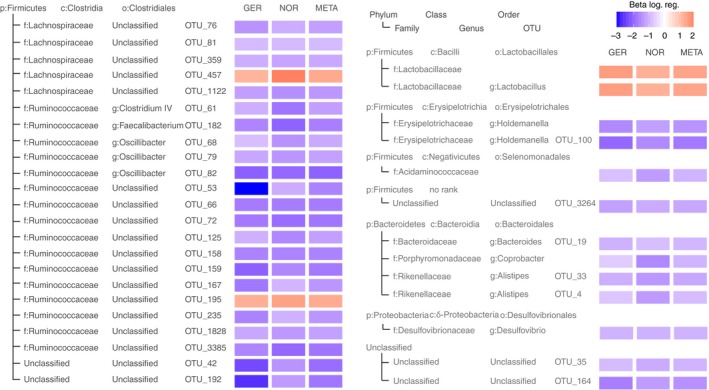
Robust results of the logistic regression within cohorts and the meta‐analysis testing for differential prevalence of taxonomic groups in PSC patients and healthy controls. Only taxa with *P* < 0.05 in each cohort, *Q*_META_ < 0.05 and concordant effect direction are shown. Colour saturation expresses the effect size (Beta) of the association. Beta‐values larger than zero (red boxes) represent a higher prevalence in PSC patients, taxa with values less than zero (blue) are less prevalent in PSC patients. Details on the model coefficients and the resulting *P*‐values in the cohorts and the meta‐analysis can be found in Table S4. p: phylum, c: class, o: order, f: family, g: genus

In the next step, we aimed to discover new robust taxonomic distribution patterns between patients with PSC and controls across all taxonomic hierarchy levels. A detailed summary is provided in Figures 2 and 3 as well as Tables S3 and S4.

A total of 20 taxa in the German and 18 taxa in the Norwegian cohort showed altered (continuous) abundance in patients with PSC. However, only seven of these met the meta‐analysis criteria (see Section 2).

Robust and cohort‐spanning increased relative abundances in patients with PSC were displayed by the phylum Proteobacteria, represented by the class Gammaproteobacteria, order Lactobacillales and the class Bacilli (all *Q*_META_ < 0.0001, respectively). An OTU belonging to the genus *Coprococcus* was the only taxon with cohort‐consistent decreased abundance in PSC (*Q*_META_ = 0.017). For the differentially abundant taxa, an additional analysis was performed in the German cohort, to assess whether these signals are truly driven by disease or may be influenced by dietary differences. Only for the class Bacilli and order Lactobacillales a significant influence of protein intake could be observed (both *P* = 0.03), this however, did not influence the strongly significant signals of disease association (*P* = 1.1 × 10^─7^ and *P* = 8.9 × 10^─8^, respectively).

Regarding microbial (binary) prevalence patterns, we observed an extensive depletion in patients with PSC compared to controls affecting 32 taxa. Among these were the genera *Holdemanella* and *Desulfovibrio* as well as OTUs classified as *Faecalibacterium* and *Clostridium IV* (both *Q*_META_ < 0.0001).

### 3.5 Microbiota alterations in PSC are independent from associated colitis, medication or grade of colonic inflammation

For the analysis of effects of medication and calprotectin the same aforementioned statistical models were applied with inclusion of the respective data as additional independent variables. No differences in levels of faecal calprotectin could be found between PSC patients with and without IBD. Additionally, neither medical treatment with UDCA, 5‐ASA or Azathioprine, nor faecal calprotectin levels exhibited any effect on or correlation with the microbiota in PSC (Supporting information).

PSC‐IBD has a genetic basis and clinical phenotype different from classical UC.^9^ Therefore, PSC could drive the phenotype of intestinal inflammation, as well as intestinal microbiota composition. Therefore, we investigated if the microbiota signature in PSC‐IBD is closer to PSC only or closer to UC.

Neither in the German nor in the Norwegian cohort there was any significant difference in beta‐diversity between patients with PSC only and PSC‐IBD (*P* > 0.05, respectively). Since functionally important taxa may be differentially abundant even in the absence of significant overall beta‐diversity, we explored potential cohort‐spanning taxonomic differences between patients with PSC only and PSC‐IBD (Tables S5, S6 and S8).

Abundance‐based models comparing PSC only to PSC‐IBD showed no cohort‐spanning signals. Additionally, there were significant differences between PSC and UC in abundance and diversity, strongly indicating that PSC drives the microbiota associations observed in patients both with PSC only as well as in those with PSC‐IBD. The only robust taxonomic differences detected between PSC only and PSC‐IBD were decreased prevalences of *Bilophila* (*Q*_META_ = 0.017) and an OTU assigned to *Bacteroides* (OTU_28; *Q*_META_ < 0.0001) in patients with PSC‐IBD.

### 3.6 PSC and UC are both characterised by altered microbiota, but cannot be differentiated by single taxa

We investigated if the observed difference of beta‐diversity between patients with PSC and UC can be traced to robust differential distribution of individual taxa (Tables S9 and S10). In both cohorts, the phylum Firmicutes was significantly increased in patients with PSC compared to patients with UC (*Q*_META_ = 0.011). We found no cohort‐spanning differences of lower hierarchy level taxa except for one OTU assigned to the genus *Ruminococcus* (OTU_59; *Q*_META_ < 0.01; Figure 3).

### 3.7 The faecal microbiota profile can predict the diagnosis of PSC across different geographical regions

**Figure 4:**
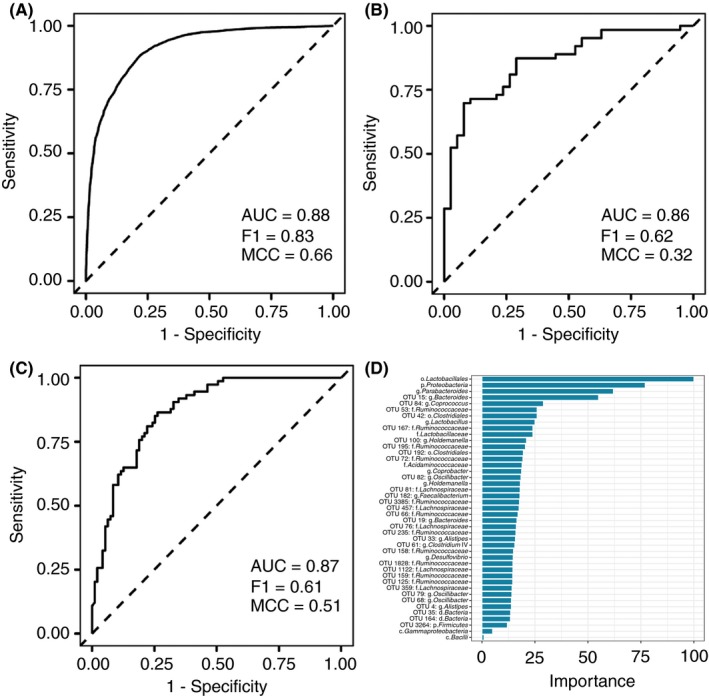
Receiver operating characteristic curve of random forest classification PSC vs controls across cohorts. Displayed are (A) the 0.632 bootstrap results from the pooled German and Norwegian cohort, (B) the classifier trained on the German cohort and validated on the Norwegian cohort and (C) vice versa. Features included in the model were the taxa with robust differential distribution between PSC and controls. (D) Feature importance of the respective taxa in the pooled classifier was ranked by Gini index

In order to investigate, whether faecal microbiota can be used to predict the presence of disease, we applied random forests classification to the pooled cohort of controls and patients with PSC (n = 270 subjects) using default hyperparameters. As baseline model variables all taxa with robust differential distribution were included (n = 43 features). Implementing 0.632 bootstrap resampling, a high performance with an average AUC of 0.88 was achieved (F1 = 0.83, MCC = 0.66; Figure 4A, taxon importance for the classifier evaluated by Gini index is displayed in Figure 4D). Training of the classifier on the German cohort and validation on the Norwegian subjects resulted in an AUC of 0.86 (F1 = 0.62, MCC = 0.32; Figure 4B). Classifier training with the Norwegian cohort and testing on the German population resulted in an AUC of 0.87 (F1 = 0.61, MCC = 0.51; Figure 4C). Further in‐depth exploration of the classification is provided in the Supporting information.

### 3.8 Diet has minor impact on microbial community alterations in PSC

Univariate comparisons of disease groups to healthy individuals showed only a minor reduction in daily water intake in PSC patients (*P*_adj_ = 0.049). Comparing multivariate differences in major dietary patterns in 220 samples of the German cohort, no significant differences were seen according to age (*P* > 0.05, *R* ^2^ = 0.002), BMI (*P* > 0.05, *R* ^2^ = 0.008) and diagnosis (*P* > 0.05, *R* ^2^ = 0.013). Gender, however, showed a strong impact (*P* < 0.001, *R* ^2^ = 0.2), as expected. Using the mentioned macronutrients and questionnaire derived intake variables as covariates in the analysis of disease‐associated shifts in microbial beta‐diversity yielded no significant associations (*P* > 0.05, respectively) and also revealed only minor effects on the still highly significant change in community composition associated with PSC (*P* = 0.003, *R* ^2^ = 0.019).

## 4 DISCUSSION

It is unclear, to which extent reported single centre microbiota studies in PSC were influenced by environmental factors and whether reported associations would remain significant across different geographical regions. In this study, we analysed the faecal microbiota of patients with PSC including patients from a German and a Norwegian cohort based on 16S rRNA gene‐amplicon sequencing profiles. To the best of our knowledge, this is the largest microbiota study focusing on PSC so far. The previously analysed cohorts^13^, ^16^ were reprocessed using a unified sequencing‐library preparation and data analysis workflow to reduce technical and statistical disparities. Controlling for cohort‐specific effects and potentially false positive results, the joint analysis of these two cohorts facilitated the identification of extensive disease‐associated changes in the faecal microbiota structure in PSC independent from geographical region. We identified several microbial signals previously not described in association with PSC, including an increased abundance of the phylum Proteobacteria, likely driven by the class Gammaproteobacteria, increased abundance of the genus *Parabacteroides*, and increase of one OTU belonging to the genus *Bacteroides.* Gammaproteobacteria comprise gram‐negative bacteria with lipopolysaccharide (LPS) containing membranes, such as Enterobacteriaceae.^25^ Multiple lines of evidence point to LPS as a common co‐factor of liver injury.^26^ Individual variation in Gammaproteobacteria has been directly linked with susceptibility to fatty liver disease.^27^ The increase in *Parabacteroides* is intriguing, as it has been demonstrated that this bile‐tolerant taxon is linked to changes in cholesterol and bile acid metabolism.^28^, ^29^

For eight taxa across different taxonomic levels we demonstrated a consistently increased abundance in patients with PSC. These findings confirm previously found associations, eg for the genera *Veillonella* 13, 16 and *Streptococcus.* 17 Increased abundance of both were previously found in patients with primary biliary cholangitis (PBC) and patients with liver cirrhosis of different origins.^12^, ^30^ This indicates that these alterations might be rather unspecific features of chronic liver disease. Additionally, we confirmed a markedly reduced abundance of one OTU belonging to the genus *Coprococcus* in patients with PSC. This taxon has previously been found to be decreased in patients with UC,^31^ which is consistent with our own findings (Supporting information). This supports the notion of *Coprococcus* as an indicator of general gut integrity. Other previously described associations of bacterial taxa with PSC could not be confirmed, such as the recently described association with the genus *Klebsiella*.^32^

We could demonstrate an extensive alteration of the microbial community structure in patients with PSC, identifying 36 taxa with differential prevalence patterns associated with the disease, of which 32 were less present in PSC patients and including the genera *Faecalibacterium* and *Clostridium IV*. Both genera comprise butyrate‐producing species, which provide an important energy source for intestinal epithelia and display an array of beneficial immunological properties.^33^ *Faecalibacterium* was attributed with beneficial immunoregulatory properties and proposed as a pharmacobiotic agent to treat inflammatory diseases.^34^ This finding further underlines its potential importance for the understanding of the pathophysiology and development of novel therapeutic approaches in inflammatory intestinal and liver diseases, where depletion of *Faecalibacterium* is a frequently observed common trait.^11^, ^30^

The results of the machine learning classification highlight that faecal microbiota can be used to detect PSC in geographically separate cohorts. This might underline the potential pathophysiological significance of the faecal microbiota and a potential clinical value as a future biomarker. While the generalisability of the model is limited by the fact that the prevalence of PSC in the general population is significantly different from the prevalence in our cohort, the significant PSC‐specific overlap of the faecal microbiota is promising. The results of the classifier additionally complement the results of the GLM‐based analysis, identifying a subset of the same taxa that were found to be significantly changed in relative abundance to also be most important (scaled Gini Index > 50) for the pooled classifier.

In previous studies of the mucosal microbiota in UC patients with and without PSC, city of origin was the main determinant of gut microbiota profile despite identical handling of all samples and no consistent differences between UC and PSC with colitis were observed.^35^ This is in line with the present findings, as significant differences were detected between PSC and UC in each cohort separately, however, only few of them were consistently observed across both cohorts. We can therefore not answer conclusively if microbiota profiling is also sufficient to distinguish PSC from UC in a geographically independent manner.

Our results strongly suggest that the increase of abundance or prevalence in PSC in contrast with controls is specific for certain taxa. Therefore, their potential role in the pathogenesis of PSC should be investigated. In addition, the extensive loss of many taxonomic groups is relatively unspecific and may potentially be explained by general effects of chronic inflammation rather than by disease‐specific etiology.

Only marginal differences were identified in our study between patients with PSC only and PSC‐IBD. This suggests that the liver disease and not the colitis is the primary driving force behind the observed gut microbial dysbiosis and that, as the differences in inflammation‐localisation in the colon already suggest, PSC‐IBD displays significant pathophysiological differences to UC.

Our study has several strengths and shortcomings. Notably, even though the German and Norwegian samples were processed with kits from different manufacturers, we could show extensive overlap in biological signals. Analysis of dietary patterns, that were available for the German cohort, showed no general differences between individuals based on disease status. Only minor influences on beta diversity were observed, which did not affect the clear disease‐associated shift in microbial communities. Previous studies investigating the influence of dietary patterns on the microbiota could show, that indeed diet can influence microbiome compostion.^36^, ^37^ However, these were performed on larger study populations (*n* > 1000) and still only found small individual effects, thus to really investigate diet‐disease interactions, larger, well‐typed PSC cohorts are needed, which are currently not available.

Although facilitating large‐scale analysis, amplicon‐based marker gene surveys always come with trade‐offs regarding fragment‐specific biases in amplification and taxonomic assignment quality, which may in part explain differences between studies. Ultimately, the intestine must be regarded as an open, highly dynamic and spatially heterogeneous system, and faecal samples can only serve as a proxy for the actual state. Nevertheless, we here present the largest microbiota study performed in PSC to date and show robust and geography‐spanning alterations in the faecal microbiota, in spite of sampling and technical differences between the centres. This strongly supports the conclusion that the observed microbiota changes are disease specific, and not primarily driven by environmental factors.

In summary, patients with PSC from different geographical regions display shared differences in gut microbial composition compared to healthy controls, independent from the presence of concurrent IBD and the use of common medication. The PSC specific gut microbiota signature might have diagnostic potential and provides a strong rationale for further microbiome meta‐analyses with even larger international cohorts. These should also utilise metagenomic sequencing, profiling of microbial metabolites as well as longitudinal designs to further define the role of the gut microbiota in the pathogenesis and clinical care in PSC. Furthermore, additional patients with chronic or cholestatic liver disease should be included for validation of disease specific effects. This study focused on broad, disease‐specific signals, as these are likely to be directly connected to the largely unknown etiology of PSC, independent of environmental influences. Ultimately, this may lay the foundation for new therapies targeting the gut microbiota in PSC.

## AUTHORSHIP

*Guarantor of the article:* None.

*Author contributions:* CS and AF designed the study and obtained funding. RZ, MKu, JH, WL, MT, TK, AWL, GD, FL and MKr acquired and quality‐controlled patient samples and data. F‐AH supervised sample processing and sequencing. MR and TL performed statistical analyses. MR, F‐AH, TL, CB, RZ, MKu, LT, JH, CS and AF interpreted data and drafted the manuscript with input and critical revision from all authors. All authors revised and approved the final version of the manuscript.

## APPENDIX 1 The authors’ complete affiliation list

Malte Rühlemann, Femke‐Anouska Heinsen, Corinna Bang, Louise Thingholm, and Andre Franke, Institute of Clinical Molecular Biology, Christian‐Albrechts‐University of Kiel, Kiel, Germany; Timur Liwinski, Roman Zenouzi, Ansgar Lohse, and Christoph Schramm, Department of Medicine, University Medical Center Hamburg‐Eppendorf, Hamburg, Germany; Martin Kummen, Tom Karlsen, and Johannes Hov, Norwegian PSC Research Center, Division of Surgery, Inflammatory Medicine and Transplantation, Oslo University Hospital Rikshospitalet, Oslo, Norway and Research Institute of Internal Medicine, Oslo University Hospital Rikshospitalet, Oslo, Norway; Marie Tempel and Wolfgang Lieb, Institute of Epidemiology, Christian‐Albrechts‐University of Kiel, Kiel, Germany; Gerald Denk, Department of Medicine II, Liver Center Munich, University Hospital, LMU Munich, Munich, Germany; Frank Lammert and Marcin Krawczyk, Department of Medicine II, Saarland University Medical Center, Saarland University, Homburg, Germany; Marcin Krawczyk, Laboratory of Metabolic Liver Diseases, Center for Preclinical Research, Department of General, Transplant and Liver Surgery, Medical University of Warsaw, Warsaw, Poland; Christoph Schramm, Martin Zeitz Centre for Rare Diseases, University Medical Center Hamburg‐Eppendorf, Hamburg, Germany
