# Supplementary material for: Consistent alterations in faecal microbiomes of patients with primary sclerosing cholangitis independent of associated colitis
Source: Aliment Pharmacol Ther. 2019 Jun 28;50(5):580–9. doi: 10.1111/apt.15375 (PMC6899739; doi:10.1111/apt.15375)
Supplement: Supplementary file 2 [file APT-50-580-s002.docx]

**SUPPLEMENTARY MATERIAL: Consistent Alterations in Fecal Microbiomes of Patients With Primary Sclerosing Cholangitis**

Malte Rühlemann^1,#^, Timur Liwinski^2,#^, Femke-Anouska Heinsen^1,#^, Corinna Bang^1^, Roman Zenouzi^2^, Martin Kummen^3,4^, Louise Thingholm^1^, Marie Tempel^5^, Wolfgang Lieb^5^, Tom Karlsen^3,4,6,7^, Ansgar Lohse^2^, Johannes Hov^3,4,6,7^, Gerald Denk^8^, Frank Lammert^9^, Marcin Krawczyk^9,10^, Christoph Schramm^2,11, †^ , Andre Franke^1, †^

^1^ Institute of Clinical Molecular Biology, Christian-Albrechts-University of Kiel, Kiel, Germany

^2^ I. Department of Medicine, University Medical Center Hamburg-Eppendorf, Hamburg, Germany

^3^ Norwegian PSC Research Center, Division of Surgery, Inflammatory Medicine and Transplantation, Oslo University Hospital Rikshospitalet, Oslo, Norway

^4^ Research Institute of Internal Medicine, Oslo University Hospital Rikshospitalet, Oslo, Norway

^5^ Institute of Epidemiology, Christian-Albrechts-University of Kiel, Kiel, Germany

^6^ Institute of Clinical Medicine, University of Oslo, Oslo, Norway

^7^ Section of Gastroenterology, Department of Transplantation Medicine, Division of Surgery, Inflammatory Diseases and Transplantation, Oslo University Hospital Rikshospitalet, Oslo, Norway

^8^ Department of Medicine II, Liver Center Munich, University Hospital, LMU Munich, Munich, Germany

^9^ Department of Medicine II, Saarland University Medical Center, Saarland University, Homburg, Germany

^10^ Laboratory of Metabolic Liver Diseases, Center for Preclinical Research, Department of General, Transplant and Liver Surgery, Medical University of Warsaw, Warsaw, Poland

^11^ Martin Zeitz Centre for Rare Diseases, University Medical Center Hamburg-Eppendorf, Hamburg, Germany

^#^ MCR, TL and F-AH contributed equally to this work; ^†^ AF and CS jointly supervised this work

**TABLE OF CONTENTS**

**Supplementary Methods:** Pages 1 - 4

**Supplementary Results:** Pages 5 - 7

**Supplementary Tables S1,S2, and S13-S15:** Pages 7 - 12; Large supplementary tables S3-S12 in ‘Supplementary_Tables_S3-S12.xlsx’ file

**Supplementary Figures S1-S10:** Pages 13 - 21

**Supplementary References:** Page 22 - 23

**SUPPLEMENTARY METHODS**

**Sample selection and exclusion**

All individuals underwent extensive screening for confounding factors. Any signs of systemic disease assessed by routine laboratory parameters deviating from healthy reference ranges, namely CRP ≤5 mg/l, glucose between 55 and 115 mg/dl, and insulin between 2.6 and 24.9 mU/l, and a comprehensive questionnaire covering self-reported status on disease (diabetes, cancer, respiratory-, liver, coronary heart disease, heart attack, neuropathy, IBD, IBS, chronic diarrhea, asthma, organ transplantation, phlebitis, varices, venous insufficiency) were criteria for study exclusion. All study participants were unrelated individuals.

To avoid confounding by unspecific microbiota changes in chronic liver disease, PSC patients with concurrent signs of autoimmune hepatitis were excluded from the analysis. The vast majority of PSC samples did not exhibit any signs of liver cirrhosis or impaired liver function. UC was diagnosed based on medical history and colonoscopy with biopsy according to recent guidelines and excluding infectious colitis.^S1^

Exclusion criteria for all groups were treatment with antibiotics within six weeks before stool collection, missing data on age, gender or BMI, other chronic liver diseases and prior colectomy. Individuals with Crohn’s disease-like inflammatory patterns affecting the small bowel or undetermined type of colitis were excluded from the analysis due to small sample numbers in the German cohort (n=7 and n=2, respectively).

**Stool Biomarkers**

Calprotectin (as an indicator of intestinal inflammation) was measured in all German fecal samples using the Bühlmann fCAL™ ELISA kit (BÜHLMANN LABORATORIES AG) and analyzed using the SoftMax Pro Software (Molecular Devices).

**Sequencing and bioinformatics processing of 16S rRNA gene libraries**

The 16S rRNA gene library generation and sequencing was performed as previously described.^S2^ In short, the V1-V2 region of the 16S rRNA gene was sequenced on the MiSeq platform, using the 27F-338R primer pair and a dual indexing approach. Obtained sequences data were trimmed in paired-end mode using sickle and default parameters.^S3^ Forward and reverse reads were merged using VSEARCH v2.5 and quality controlled using FastX-Toolkit. Sequences with more than 5% per-base-quality below 30 were discarded. Chimeric reads were identified by both a reference-based (utilizing the UCHIME ‘gold.fa’ database obtained from https://drive5.com/uchime/uchime_download.html) and a de-novo approach based on the UCHIME algorithm implemented in VSEARCH.^S4^ Sequences were taxonomically annotated using the SINTAX algorithm in USEARCH (v9) and the RDP database version 14.^S5^ Sequences that could not be assigned to the domain ‘Bacteria’ were discarded. Clustering into Operational Taxonomic Units (OTUs) was performed using the VSEARCH. Sequencing-depth was normalized by randomly picking 10.000 reads per sample and abundance tables for all taxonomic levels and OTUs were generated. The number of 10,000 sequences was choses, as this was defined as the minimum sequence count threshold after QC and a widely used choice for human gut microbiome analysis.

**Analysis of differences in alpha- and beta-diversity**

Differences in alpha-diversity were assessed using the non-parametric Wilcoxon rank-sum test. Permutational MANOVA (*adonis* function from the *vegan* package^S6^) using Bray-Curtis dissimilarity was applied to assess differences in correcting for the covariates age, gender and BMI. Unconstrained ordination plots were created based on Bray-Curtis dissimilarity (*vegdist* function in the *vegan* package). Square-root transformation of the dissimilarity was used to eliminate negative eigenvalues of ordination axes.^S7^

**Analysis of healthy core microbiota**

To explore the overlap and differences in healthy microbiota between the Norwegian and the German cohorts, core microbiota analysis was performed. A Venn diagram was drawn to illustrate common taxa prevalence on the genus taxonomic level (“vennDiagram” R package^S8^). To avoid bias by rare taxa, only taxa present in at least 5% prevalence in each respective cohort were considered. Core microbiota heatmaps were drawn separately and compared for the Norwegian and German healthy cohorts as described previously,^S9^ considering genera with minimum prevalence of 10% in the respective cohorts.

**Selection of genera from Kummen *et al.* and Sabino *et al.* for replication**

In the article by *Kummen et al.*^S10^, Figure 3 summarizes 10 taxonomic groups as differentially abundant in PSC compared to HC. Of these, five were classified on genus level. The genus *Desulfovibrio* was not present in any of the two cohorts, thus discarded from the analysis. All members of the family Christensenellaceae were classified down to genus level *Christensenella,* perfectly correlating to the genus level abundance, thus being included in the analysis. Sabino *et al*.^S11^ identified four genera as “PSC signature” (*Fusobacterium, Enterococcus, Streptococcus* and *Lactobacillus*), as summarized in their Figure 3, all of which could be recovered in both cohorts presented in this study.

**Analysis for differential prevalence and abundance patterns and meta-analysis**

All taxa with at least 20% presence were transformed to dichotomous features (0 for absence, 1 for presence) and subjected to logistic regression (R base function *glm)*, adjusting for the covariates age, gender and BMI. If the abundance in the samples where the taxon was present exceeded a mean abundance of 0.5% in one of the groups being compared, the taxa were additionally subjected to abundance-based analysis using hurdle models as implemented in the *pscl* R package ^S12^ if the respective taxa contained zero values or otherwise generalized linear models (GLM) as provided in the *MASS* R package.^S13^ Both models were applied assuming a negative binomial distribution for the count part of the abundances and including the covariates age, gender and BMI analogously to the logistic regression. Extreme abundance values deviating more than five interquartile ranges from the group median were excluded to minimize outlier-driven false positive results. Count models give more statistical power to detect differential expression than approximate normal models in high-throughput sequencing data.^14^ Therefore, high-throughput sequencing datasets were advised to be treated as count data.^15^ Moreover, count models have the advantage for properly separating biological from technical variation.^16^ The meta-analysis was performed separately for logistic regression and abundance-based models, however following the same approach. Briefly, this approach combines the effect sizes from the models combined weighted by a termed based on the inverted variance as calculated from the standard errors taken from the respective models. The Z-score resulting from this model can be used to calculate the meta-analysis P-value. These P-values were subsequently corrected for multiple testing using the Benjamini-Hochberg-correction. Only taxa with a resulting P<0.05 in each cohort and Q_META_<0.05, after correction across all taxonomic levels used in the analysis to ensure a minimum of false postitves, were regarded as robustly differentially distributed.

**Analysis of effects of medical treatment**

To assess the effects of PSC medication on the microbiota, all medications received by at least 10% of PSC patients were tested. These included ursodeoxycholic acid (UDCA), mesalazine (5ASA) and azathioprine (AZA). Analyses of effects on beta-diversity and single taxonomic groups were performed as described earlier, using intake of the respective drugs as additional covariates.

**Machine learning classification**

Random forest classification was implemented using the package *ranger* ^S17^ via the *caret* interface with default hyperparameters. The number of variables at each node (mtry) was held constant according to the square root of included model features as suggested by Breiman.

Taxa with robust differential distribution were included either as continuous variables or dichotomous features, according to the prespecified abundance threshold.

To estimate the generalization error the 0.632 bootstrap estimator was applied with 999 iterations. ^S18^ For Evaluation of model performance, the area under the receiver operating characteristic (ROC) curve (AUC) was used. In addition, contingency table related measures are reported: The F1 score represents the harmonic average between the recall (sensitivity) and precision (true positive rate) of a model. The optimal value is reached at F1=1. Matthews Correlation coefficient (MCC) reflects the improvement of agreement between predicted and actual values over random prediction with respect to frequency of each class. ^S19^ Therefore, the MCC represent a performance measure which is robust to class imbalance in a data set. Perfect agreement is achieved at MCC=1.

Feature importance of the pooled random forest classifier was calculated using the Gini index (scaled 0 to 100).

Learning curves were drawn to explore the relationship between the size of the training cohort and classifier performance.

To validate the resampling results of the pooled random forest classification, additional resampling methods were implemented: repeated cross validation (3 folds, 10 repeats) and leave one out cross-validation. ^S20^

To proof that the classification performance is better explained by the data structure than the chosen model, we implemented further independent machine learning algorithms: (guided) regularized random forest, radial kernel support vector machine and extreme gradient boosting. ^S21-S23^ Generalization error of each model was estimated using 0.632 bootstrapping.

To control for potential overfitting due to many features, we used two different feature reduction strategies: First, we implemented recursive feature elimination using repeated cross-validation (3 folds, 10 repeats). The dependence of ROC and MCC on the number of features was assessed visually. Second, we applied the ‘Boruta’ feature selection algorithm with default parameters.^S24^ Within this framework, so called ‘shadow variables’ are created via permutation of the original variables. The most important variables are selected via comparison of the importance of each real variable with the maximum value of all shadow variables. The pooled random forest classification was repeated with a reduced feature number selected by the Boruta algorithm using 0.632 bootstrapping.

**Analysis of calprotectin measures**

Differences in fecal calprotectin were assessed using the non-parametric Wilcoxon rank-sum test (*wilcox.test* from the R-base package).

**SUPPLEMENTARY RESULTS**

**Healthy Norwegian and German cohorts share similar core microbiota**

Of the 144 genera present in at least 5% of either the Norwegian or the German cohort, 122 (84.72%) were prevalent in both cohorts (**Figure S1**). Only six genera (4.16%) were found exclusively in Norwegian patients. Exclusive taxa were confined to low abundance genera (3^rd^ quartile of mean relative abundance 3.49×10^-5^). Core microbiota heatmaps showed similar taxonomic abundance/prevalence patterns in both cohorts (**Figure S2**).

Furthermore, applying principal coordinates analysis to the healthy cohorts, the first principal coordinate, which showed the most pronounced difference between Norwegian and German healthy controls (Wilcoxon rank-sum test; p=1.89×10^-4^) was highly correlated with the Shannon index (Spearman’s rank correlation test; r=0.53, p=6.23×10^-11^; **Figure S3**).

To summarize, Norwegian and German control cohorts share similar healthy core microbiota, differences in beta-diversity are mainly explained by the difference in alpha-diversity, and to a lesser extent by disparities of the taxonomic composition.

**Minor differences between German and Norwegian healthy controls**

Four genera (*Blautia* (P_adj_=4x10^-4^, β=-1.04), *Escherichia/Shigella* (P_adj_=9.5x10^-3^, β=-4.27)*, Akkermansia* (P_adj_=8x10^-4^, β=-3.06) and *Haemophilus* (P_adj_=1.3x10^-2^, β=-2.8)) as well as the family *Enterobacteriaceae* (P_adj_=3x10^-4^, β=-3.45) were significantly higher abundant in the German samples compared to the Norwegian samples, whereas the genus *Clostridium Cluster IV* (P_adj_=8.9x10^-3^, β=1.18) and the class Alphaproteobacteria (P_adj_=1.3x10^-3^, β=2.53) showed higher abundances in the Norwegian cohort. Additionally, the *Escherichia/Shigella* cluster was more prevalent in the German cohort (P_adj_=1.21x10^-2^, β=-1.89).

**Medical treatment of PSC has no influence on the fecal microbiome.**

Initiation of UDCA therapy has previously been reported to change the intestinal microbiome of patients with primary biliary cirrhosis (PBC).[19] In our cross-sectional cohort of PSC patients, neither UDCA, nor 5ASA and AZA had any significant influence on the overall beta-diversity (P>0.05) or individual taxa (all P_META_>0.05).

**Calprotectin not significantly elevated in PSC**

Pairwise Wilcoxon-rank-sum-test of fecal Calprotectin measurements did not show any differences between PSC patients and healthy controls (P_Wilcox,controls-PSC_=0.97), however UC patients differed from both PSC and controls (P_Wilcox, controls-UC_=0.0077, P_Wilcox,PSC-UC_=0.02). Splitting PSC patients into PSC-only and PSC-IBD, the test is significant between PSC-only and UC (P_Wilcox,PSConly-UC_=0.013), however neither between PSC-only and PSC-IBD (P_Wilcox,PSConly-PSC-IBD_=0.48) and PSC-IBD and UC (P_Wilcox,PSC-IBD,UC_=0.12).

**Known patterns in the microbiota of UC compared to healthy controls.**

We observed major differences in the microbiota of patients with UC and controls and were able to reproduce results of former studies.

Patients with UC displayed a cohort-spanning increase in bacteria belonging to the phylum of Proteobacteria and more specifically the class Gammaprotebacteria (Q_META_=1.3x10^-7^, P_GER_=4.7x10^-6^, P_NOR_=5.7x10^-5^ and Q_META_=7.7x10^-3^, P_GER_=8.3x10^-3^, P_NOR_=3.6x10^-2^). Additionally, we could observe an increase in *Pasteurellacae* (Q_META_=1.8x10^-2^, P_GER_=3.8x10^-2^, P_NOR_=3.3x10^-2^) and OTU_13 belonging to the genus *Bacteroides* (Q_META_=1.0x10^-4^, P_GER_=2.9x10^-4^, P_NOR_=5.8x10^-3^)*.* A decreased abundance associated with UC was found in the phylum Firmicutes (Q_META_=1.0x10^-4^, P_GER_=2.9x10^-4^, P_NOR_=5.8x10^-3^) and in sub-clades thereof. The strongest effects were seen in two OTUs, OTU_84: *Coprococcus spp.* (Q_META_=1.0x10^-4^, P_GER_=4.8x10^-5^, P_NOR_=1.8x10^-2^) and OTU_3385 belonging to the family of *Ruminococcaceae* (Q_META_=1.0x10^-4^, P_GER_=9.8x10^-3^, P_NOR_=6.4x10^-3^, **Figure S4**, **Supplementary Table S7**).

Furthermore, we found a higher prevalence of known mediators like *Akkermansia*, *Bilophila*, *Coprococcus* and *Ruminococcus* in healthy controls, which were among a total of 47 taxonomic groups more likely to be absent in UC cases (**Supplementary Table S8**, **Figure S5**), also including four OTUs belonging to the genus *Faecalibacterium*. *Bilophila* was also found to be reduced in PSC patients, indicating an association to overall gut health. Only three taxa were found to be more prevalent in UC patients, three of which were unspecified members of the family *Lachnospiraceae* (OTU_119, OTU_376 and OTU_457) and one classified as *Clostridium Cluster XVIII spp.* (OTU_109).

**Machine learning results**

A detailed summary of the cross-cohort classification is provided in **Table S13**. Ranked Gini variable importance of the pooled random forest classifier is illustrated in **Figure S6**.

Learning curves of the random forest classifier (**Figure S6**) indicate a constantly perfect classification of the training set (regardless of the size). The baseline test performance is high already at small training set sizes (AUC>0.8) and converges with the training performance with increasing training sample sizes (AUC>0.9).

Repeating the pooled random forest classification over more conservative resampling methods resulted in a less optimistic but still high classifier performance (**Table S14**).

Repeating the pooled classification (n=270 subjects, n=43 features) with different models resulted in a comparably high performance (**Table S15**).

Implementing recursive feature elimination, random forest classifier performance steeply increased with increasing feature number and plateaued at ca. 20 features (**Figure S7**).

Boruta feature selection algorithm identified 23 variables which significantly contributed to loss random forest classifier performance (**Figure S8**). Repeating the classification with the reduced variables set did not result in a loss of classifier performance (AUC=0.89, F1=0.83, MCC=0.67; **Figure S9**). The selected taxa are shown in **Figure S8** ranked by Gini importance (scaled 0 to 100).

**SUPPLEMENTARY TABLES**

**Supplementary Table S1:** Summary of the targeted analysis of previously identified genera associated with PSC. Only genera that could be recovered in the dataset are presented. Mean abundance is calculated based on non-zero values. Details of models and P-values in the cohorts and the meta-analysis can be found in Supplementary Table S2.

| **Differential prevalence** | | | |  | |  | | |  | | | |  | | | |  | | |  | |
| --- | --- | --- | --- | --- | --- | --- | --- | --- | --- | --- | --- | --- | --- | --- | --- | --- | --- | --- | --- | --- | --- |
|  |  | | Prevalence (%) | | | | | | | | | | |  | | | |  | | |  |
|  |  | | GER | | | | | NOR | | | | | | | Results Logistic Regression | | | | | | |
| Genus | | Controls | | PSC | | Controls | | | | | PSC | | GER | | | | NOR | | | META | |
| Kummen et al. | |  | |  | |  | | | | |  | |  | | | |  | | |  | |
|  | *Christensenella* | | 18.9 | | 18.9 | | 18.4 | | | | 9.5 | | - | | | | - | | | - | |
|  | *Coprococcus* | | 88.4 | | 67.6 | | 97.4 | | | | 85.7 | | * | | | | n.s. | | | n.s. | |
|  | *Phascolarctobacterium* | | 46.3 | | 32.4 | | 68.4 | | | | 41.3 | | n.s. | | | | ** | | | n.s. | |
|  | *Succinivibrio* | | 0 | | 0 | | 2.6 | | | | 1.5 | | - | | | | - | | | - | |
|  | *Veillonella* | | 70.5 | | 75.7 | | 60.5 | | | | 73 | | n.s. | | | | n.s. | | | n.s. | |
| Sabino et al. | |  | |  | |  | | | | |  | |  | | | |  | | |  | |
|  | *Enterococcus* | | 13.7 | | 33.8 | | 2.6 | | | | 19 | | *** | | | | * | | | *** | |
|  | *Fusobacterium* | | 10.5 | | 20.3 | | 7.9 | | | | 17.5 | | n.s. | | | | n.s. | | | n.s. | |
|  | *Lactobacillus* | | 37.9 | | 77 | | 26.3 | | | | 55.6 | | *** | | | | ** | | | *** | |
|  | *Streptococcus* | | 91.6 | | 97.3 | | 92.1 | | | | 96.8 | | n.s. | | | | n.s. | | | n.s. | |
|  |  | |  | |  | |  | | | |  | |  | | | |  | | |  | |
| **Differential abundance** | | | |  | |  | | | |  | |  | | | |  | | |  | | |
|  |  | | Mean Abundance (%) | | | | | | | | | | |  | | | |  | | |  |
|  |  | | GER | | | | | NOR | | | | | | | Results GLM | | | | | | |
| Genus | | Controls | | PSC | | Controls | | | | | PSC | | GER | | | | NOR | | | META | |
| Kummen et al. | |  | |  | |  | | | | |  | |  | | | |  | | |  | |
|  | *Christensenella* | | 0.02 | | 0.02 | | 0.01 | | | | 0.01 | | - | | | | - | | | - | |
|  | *Coprococcus* | | 0.43 | | 0.34 | | 1.1 | | | | 0.54 | | n.s. | | | | n.s. | | | n.s. | |
|  | *Phascolarctobacterium* | | 3.26 | | 2.95 | | 1.91 | | | | 1.46 | | n.s. | | | | n.s. | | | n.s. | |
|  | *Succinivibrio* | | 0 | | 0 | | 7.99 | | | | 0.06 | | - | | | | - | | | - | |
|  | *Veillonella* | | 0.16 | | 1.8 | | 0.12 | | | | 0.42 | | * | | | | ** | | | *** | |
| Sabino et al. | |  | |  | |  | | | | |  | |  | | | |  | | |  | |
|  | *Enterococcus* | | 0.04 | | 0.51 | | 0.01 | | | | 0.31 | | - | | | | - | | | - | |
|  | *Fusobacterium* | | 0.03 | | 0.15 | | 0.01 | | | | 0.13 | | - | | | | - | | | - | |
|  | *Lactobacillus* | | 0.16 | | 1 | | 0.11 | | | | 1.01 | | * | | | | n.s. | | | n.s. | |
|  | *Streptococcus* | | 0.3 | | 0.95 | | 0.17 | | | | 0.46 | | *** | | | | *** | | | *** | |

**n.s.: not significant; * P<0.05; ** P<0.01; *** P<0.001; - : not tested due to low prevalence or abundance**

**Supplementary Table S2**: Targeted analysis of previously identified genera associated with PSC. Differential-abundance and differential-prevalence analysis was performed using generalized linear models on the non-zero count values and presence/absence status, respectively. Meta-analysis was performed using the inverse-variance weighted Z-score.

| **Differential abundance** | |  |  |  |  |  |  |  |
| --- | --- | --- | --- | --- | --- | --- | --- | --- |
| **GER** | BETA | SE | Z | P-value | Excl. Thresh | n Excluded | n Count | n Zero |
| g.Veillonella | 1,0478887 | 0,520233 | 2,0142679 | 0,04398141 | 106 | 19 | 104 | 46 |
| g.Phascolarctobacterium | 0,2685215 | 0,4539669 | 0,5915002 | 0,554185327 | 2600 | 1 | 67 | 101 |
| g.Coprococcus | -0,3091227 | 0,2698947 | -1,1453453 | 0,252066139 | 220,25 | 4 | 130 | 35 |
| g.Lactobacillus | 1,2129193 | 0,5449075 | 2,2259177 | 0,026019692 | 181 | 11 | 82 | 76 |
| g.Streptococcus | 0,8866685 | 0,235146 | 3,7707147 | 0,000162781 | 293 | 4 | 155 | 10 |
|  |  |  |  |  |  |  |  |  |
| **NOR** | BETA | SE | Z | P-value | Excl. Thresh | n Excluded | n Count | n Zero |
| g.Veillonella | 1,23446171 | 0,4553572 | 2,71097452 | 0,006708578 | 74 | 4 | 65 | 32 |
| g.Phascolarctobacterium | -0,25765376 | 0,4210821 | -0,6118848 | 0,540614 | 1686,25 | 0 | 52 | 49 |
| g.Coprococcus | -0,18317647 | 0,2755271 | -0,66482204 | 0,5061643 | 330,5 | 8 | 83 | 10 |
| g.Lactobacillus | -0,08117386 | 0,9242498 | -0,08782675 | 0,9300144 | 151 | 3 | 42 | 56 |
| g.Streptococcus | 1,14899675 | 0,2450015 | 4,68975331 | 2,74E-06 | 125 | 6 | 90 | 5 |
|  |  |  |  |  |  |  |  |  |
| **META** | w GER | w NOR | SE | BETA | Z | P-value |  |  |
| g.Veillonella | 3,694913 | 4,82276 | 0,3426412 | 1,15352751 | 3,36657598 | 0,000761076 |  |  |
| g.Phascolarctobacterium | 4,852345 | 5,639835 | 0,3087217 | -0,01431215 | -0,04635941 | 0,9630238 |  |  |
| g.Coprococcus | 13,728123 | 13,172598 | 0,1928049 | -0,24745003 | -1,28342196 | 0,1993443 |  |  |
| g.Lactobacillus | 3,367863 | 1,170634 | 0,469401 | 0,87912823 | 1,8728727 | 0,06108597 |  |  |
| g.Streptococcus | 18,08526 | 16,659517 | 0,1696505 | 1,01245035 | 5,96785826 | 2,40E-04 |  |  |
|  |  |  |  |  |  |  |  |  |
| **Differential prevalence** | |  |  |  |  |  |  |  |
| **GER** | BETA | SE | Z | P-value | Excl. Thresh | n Excluded | n Count | n Zero |
| g.Veillonella | 0,08013801 | 0,3845355 | 0,2084021 | 0,834915 | 106 | 19 | 104 | 46 |
| g.Phascolarctobacterium | -0,56652463 | 0,3402311 | -1,6651172 | 0,09588939 | 2600 | 1 | 67 | 101 |
| g.Coprococcus | -1,40763613 | 0,4256146 | -3,3073021 | 0,000941992 | 220,25 | 4 | 130 | 35 |
| g.Lactobacillus | 1,55340248 | 0,3757886 | 4,1337134 | 0,0000357 | 181 | 11 | 82 | 76 |
| g.Enterococcus | 1,11313454 | 0,4070803 | 2,7344347 | 0,006248749 | NA | 0 | 38 | 131 |
| g.Fusobacterium | 0.514840 | 0.476090 | 1.555 | 0.1198 | 19 | 1 | 25 | 144 |
| g.Streptococcus | 1,22858584 | 0,8627039 | 1,4241107 | 0,1544144 | 293 | 4 | 155 | 10 |
|  |  |  |  |  |  |  |  |  |
| **NOR** | BETA | SE | Z | P-value | Excl. Thresh | n Excluded | n Count | n Zero |
| g.Veillonella | 0,4775925 | 0,4539256 | 1,052138 | 0,292736093 | 74 | 4 | 65 | 32 |
| g.Phascolarctobacterium | -1,2079426 | 0,443242 | -2,725244 | 0,006425392 | 1686,25 | 0 | 52 | 49 |
| g.Coprococcus | -1,6575232 | 1,0862435 | -1,525922 | 0,127029198 | 330,5 | 8 | 83 | 10 |
| g.Lactobacillus | 1,2504417 | 0,4637517 | 2,69636 | 0,007010181 | 151 | 3 | 42 | 56 |
| g.Enterococcus | 2,114603 | 1,0680297 | 1,97991 | 0,047713615 | NA | 0 | 101 | 88 |
| g.Fusobacterium | 1,0566854 | 0,7143608 | 1,479204 | 0,13908576 | NA | 0 | 101 | 87 |
| g.Streptococcus | 2,5288593 | 1,6838979 | 1,501789 | 0,133151621 | 125 | 6 | 90 | 5 |
|  |  |  |  |  |  |  |  |  |
|  |  |  |  |  |  |  |  |  |
| **META** | w GER | w NOR | SE | BETA | Z | P-value |  |  |
| g.Veillonella | 6,76281 | 4,8532282 | 0,2934073 | 0,2461961 | 0,8390932 | 0,401417 |  |  |
| g.Phascolarctobacterium | 8,638772 | 5,0900057 | 0,2698883 | -0,8043332 | -2,9802448 | 0,002880181 |  |  |
| g.Coprococcus | 5,520354 | 0,8475116 | 0,3962808 | -1,4408941 | -3,6360429 | 0,000276858 |  |  |
| g.Lactobacillus | 7,081296 | 4,6497429 | 0,2919657 | 1,4333202 | 4,9092083 | 0,000000914 |  |  |
| g.Enterococcus | 6,034479 | 0,8766643 | 0,3803865 | 1,2401688 | 3,2602863 | 0,001112998 |  |  |
| g.Fusobacterium | 4,411861 | 1,9595882 | 0,396169 | 0,6814887 | 1,720197 | 0,9146034 |  |  |
| g.Streptococcus | 1,34362 | 0,35267 | 0,7678032 | 1,4989213 | 1,9522206 | 0,05091201 |  |  |

**Supplementary Tables S3-S12** can be found in the attached ‘Supplementary_Tables_S3-S12.xlsx’ file

**Table S13**: Summary of cross-cohort random forest classification.

| Cohort ^a^ | | Performance measures | | | | | |
| --- | --- | --- | --- | --- | --- | --- | --- |
| Training | Testing | AUC ^b^ | Sensitivity | Specificity | Accuracy | F1 ^c^ | MCC ^d^ |
| German | Norwegian | 0.86 | 1 | 0.25 | 0.63 | 0.62 | 0.32 |
| Norwegian | German | 0.87 | 0.44 | 1 | 0.72 | 0.61 | 0.51 |

^a^ N=169 German and n=101 Norwegian individuals, n=43 features; ^b^ area under the receiver operating curve; ^c^ F1 score is the harmonic average between precision (true positive rate) and recall (sensitivity), the best performance is reached at F1=1; ^d^ Matthews Correlation Coefficient, the MCC reflects the improvement of agreement between predicted and actual values over random prediction with respect to frequency of each class, perfect agreement is achieved at MCC=1.

**Table S14**: Performance diagnostics of the random forest classification of the pooled cohort over different resampling methods.

|  | Performance measures | | | | | |
| --- | --- | --- | --- | --- | --- | --- |
| Method | AUC ^d^ | Sensitivity | Specificity | Accuracy | F1 ^e^ | MCC ^f^ |
| Boot632 ^a^ | 0.88 | 0.84 | 0.82 | 0.83 | 0.83 | 0.66 |
| CV ^b^ | 0.82 | 0.75 | 0.71 | 0.73 | 0.73 | 0.47 |
| LOOCV ^c^ | 0.81 | 0.77 | 0.71 | 0.74 | 0.75 | 0.48 |

^a^ Bootstrap 0.632; ^b^ Repeated three-fold cross-validation (10 repeats); ^c^ Leave one out cross-validation; ^d^ area under the receiver operating characteristic curve; ^e^ F1 score is the harmonic average between precision (true positive rate) and recall (sensitivity), the best performance is reached at F1=1; ^f^ Matthews Correlation Coefficient, the MCC reflects the improvement of agreement between predicted and actual values over random prediction with respect to frequency of each class, perfect agreement is achieved at MCC=1.

**Table S15**: Performance diagnostics of the classification of the pooled cohort using different models.

|  | Model performance measures ^a^ | | | | | |
| --- | --- | --- | --- | --- | --- | --- |
| Model | AUC ^e^ | Sensitivity | Specificity | Accuracy | F1 ^f^ | MCC ^g^ |
| RRF ^b^ | 0.87 | 0.82 | 0.82 | 0.82 | 0.82 | 0.64 |
| SVM ^c^ | 0.85 | 0.79 | 0.74 | 0.78 | 0.78 | 0.56 |
| Xgboost ^d^ | 0.83 | 0.78 | 0.76 | 0.77 | 0.77 | 0.54 |

^a^ Bootstrap 0.632 was used as resampling method; ^b^ (guided) regularized random forest; ^c^ radial kernel support vector machine; ^d^ extreme gradient boosting; ^e^ area under the receiver operating curve; ^f^ F1 score is the harmonic average between precision (true positive rate) and recall (sensitivity), the best performance is reached at F1=1; ^f^ Matthews Correlation Coefficient, the MCC reflects the improvement of agreement between predicted and actual values over random prediction with respect to frequency of each class, perfect agreement is achieved at MCC=1.

**SUPPLEMENTARY FIGURES**


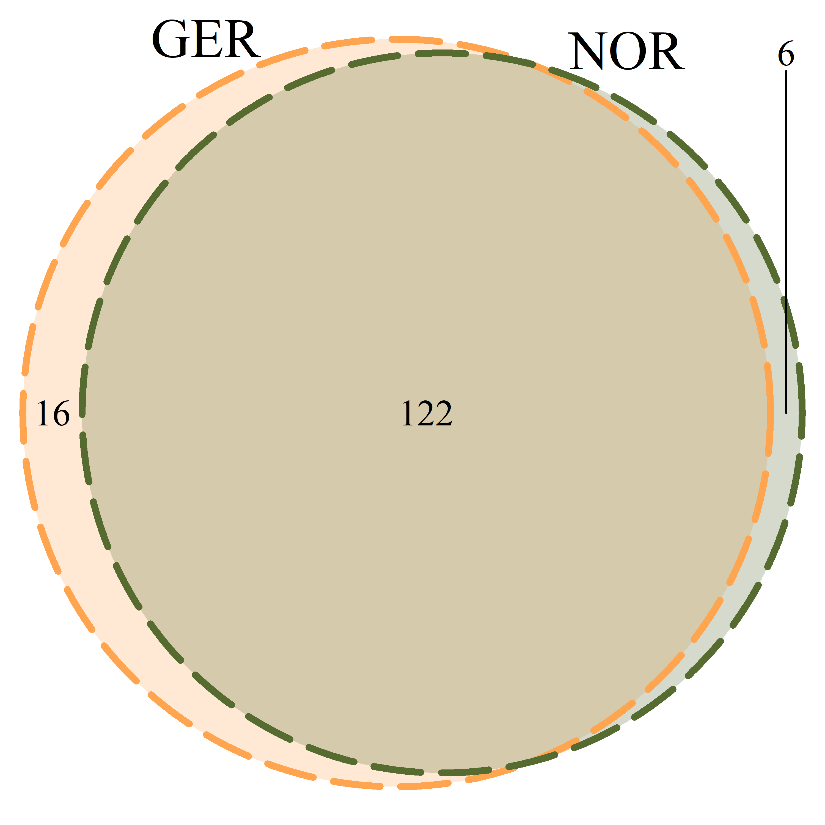


**Figure S1:** Venn diagram of co-prevalence of bacterial genera in the Norwegian (NOR) and German (GER) cohorts.


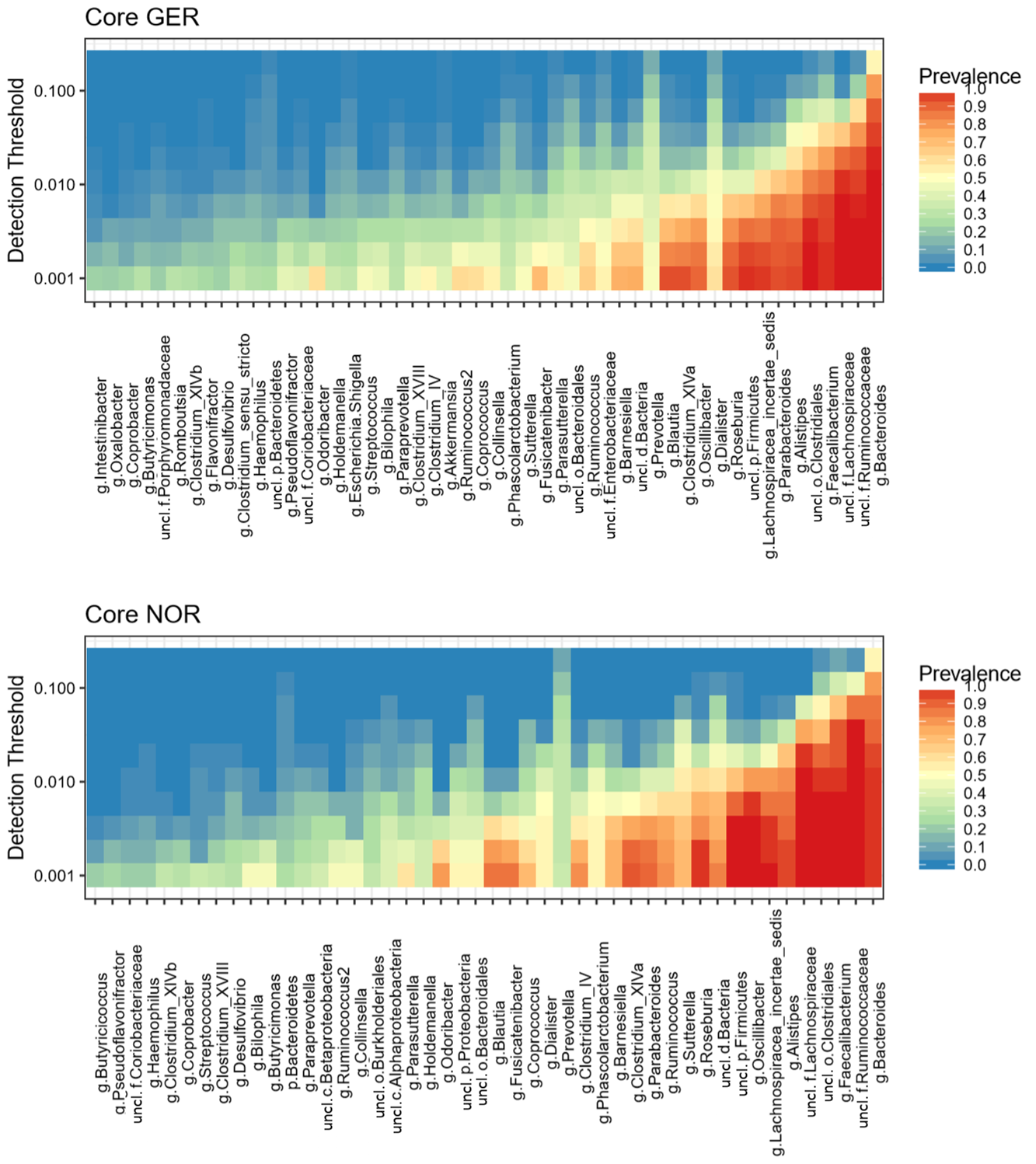


**Figure S2:** Core microbiota heatmaps illustrating prevalence rates (color gradient, red indicates ubiquitous prevalence [i.e. 1], blue indicates zero prevalence) over different detection thresholds (relative abundance) in the German (Core GER) and Norwegian (Core NOR) cohorts. Non-genus level taxa reflect community members that could only be classified to the respective taxonomic level.


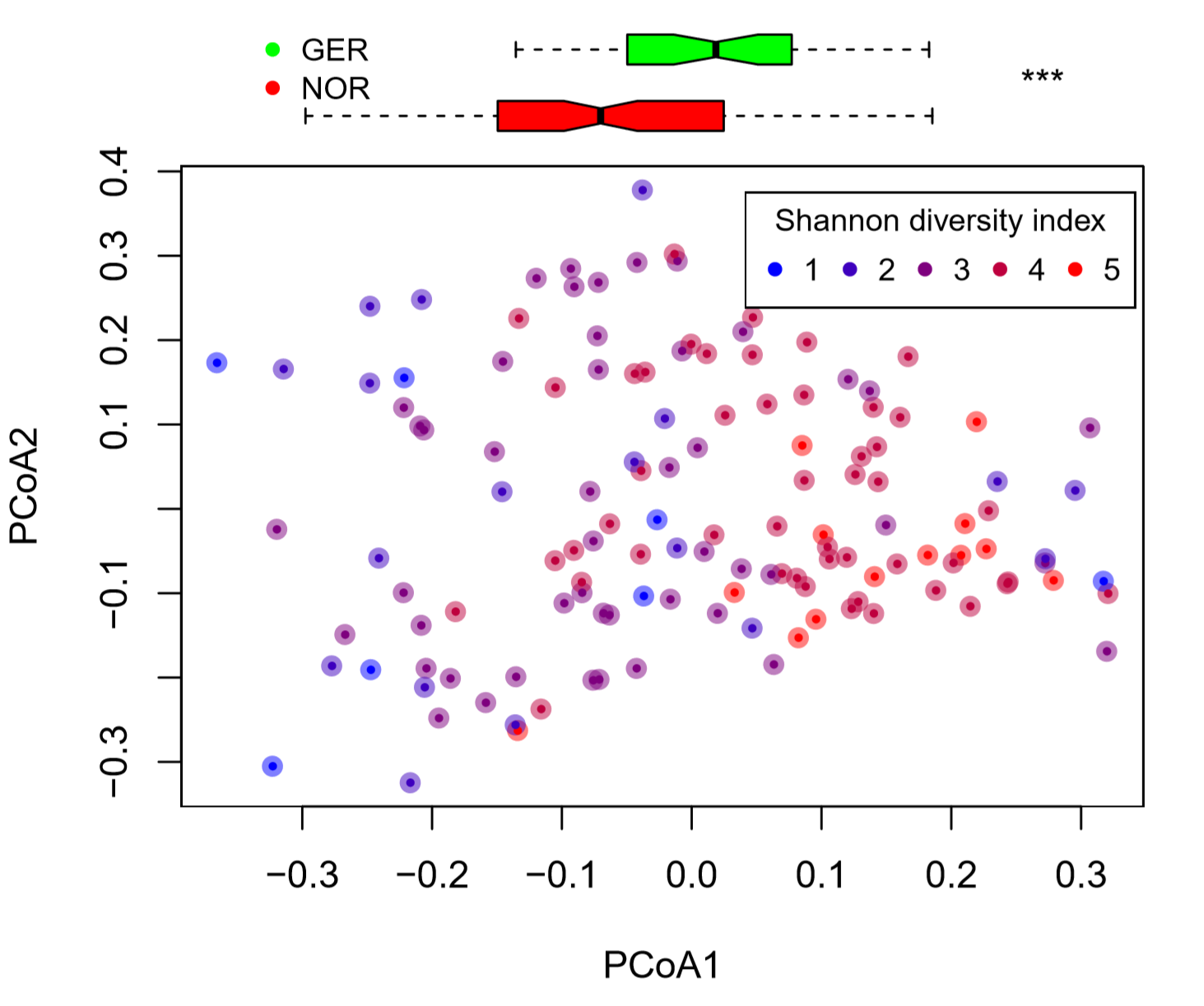


**Figure S3:** Principal coordinates analysis of fecal microbiomes of Norwegian and German healthy cohorts. The most pronounced difference between Norwegian and German healthy cohorts (boxplots) was seen on the first principal coordinate (PCoA1; Wilcoxon rank-sum test; ***: p<0.001), whereby the first principal coordinate was highly correlated with alpha diversity (color gradient according to Shannon diversity index from left to right).


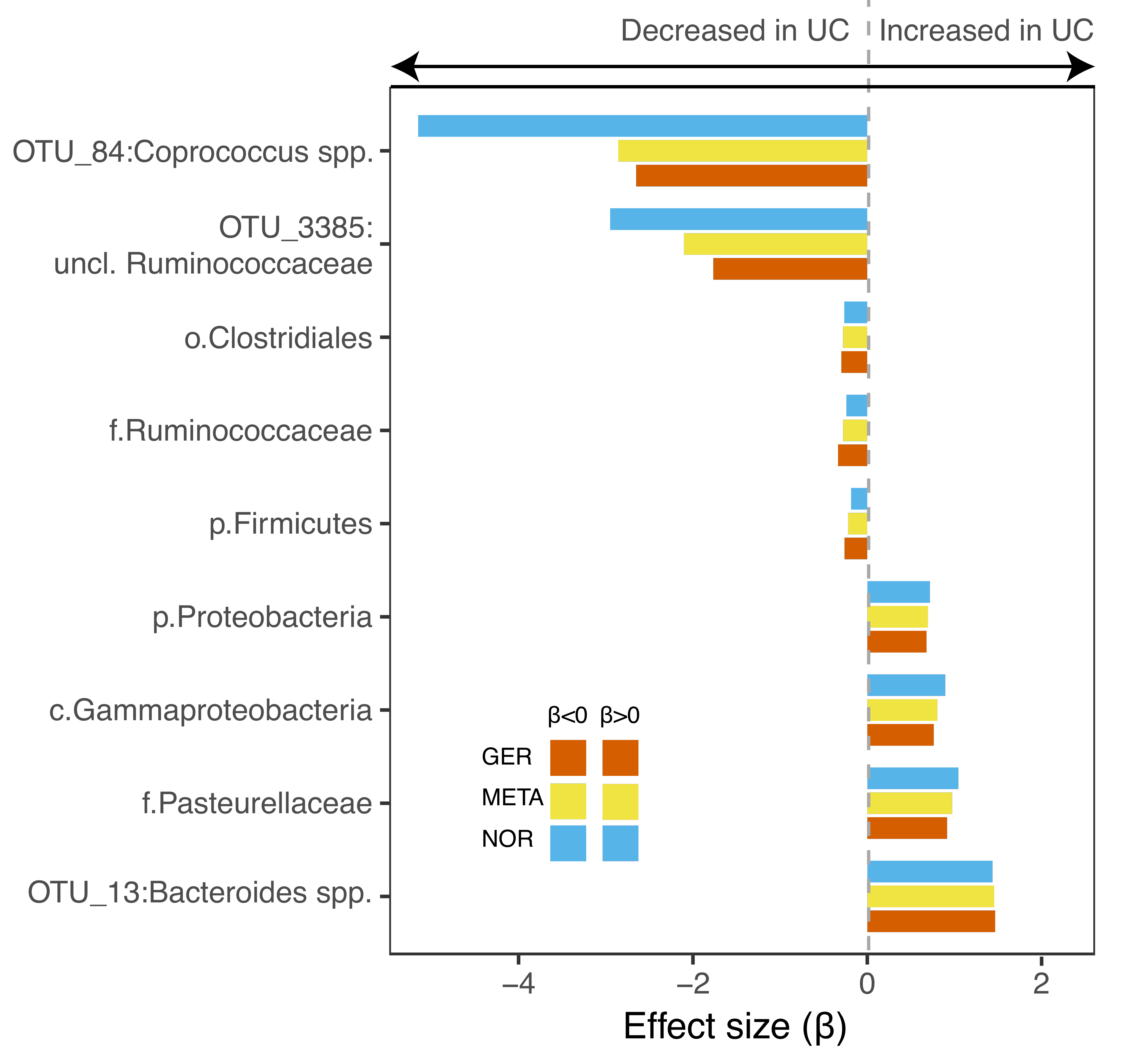


**Figure S4:** Significant and robust results of differentially abundant taxa in UC patients and healthy controls from the analysis using generalized linear models and hurdle models. Only taxa with P<0.05 in each cohort, Q_META_< 0.05 and concordant directionality are shown. Base-colors depict the effect direction (Beta) of the association. Beta-values larger than zero (red) represent a higher abundance in UC patients, taxa with values less than zero are less abundant in PSC patients. Details on the model coefficients and the resulting P-values in the cohorts and the meta-analysis can be found in **Supplementary Table S7**.


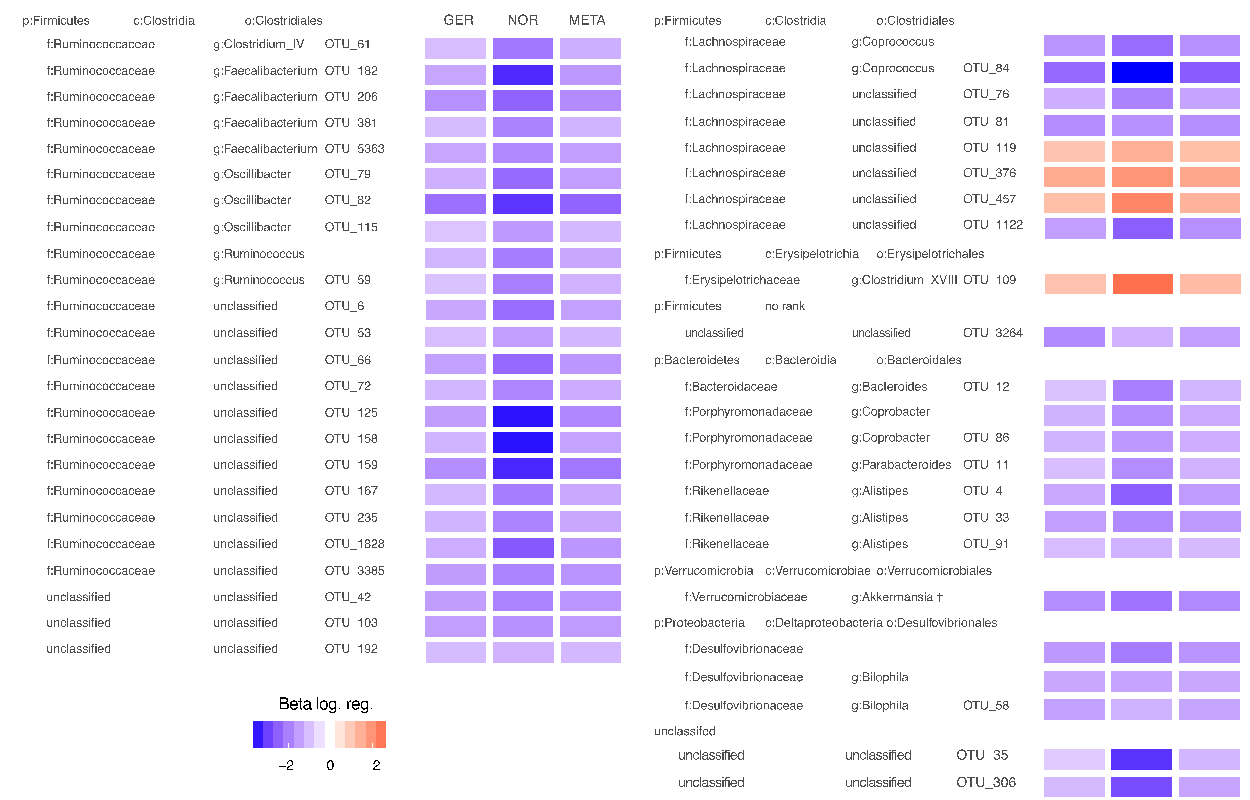


**Figure S5:** Significant and robust results of the logistic regression within cohorts and the inverse-variance weighted meta-analysis testing for differential prevalence of taxonomic groups depending on UC disease status. Only taxa with P<0.05 in each cohort, Q_META_< 0.05 and concordant directionality are shown. Colors depict the effect size (Beta) of the association. Beta-values larger than zero (red) represent a higher prevalence in UC patients, taxa with values less than zero are less prevalent in UC patients. †: Akkermansia signal could be found from phylum (Verrucomicrobia) to genus level. Details on the model coefficients and the resulting P-values in the cohorts and the meta-analysis can be found in **Supplementary Table S8**.


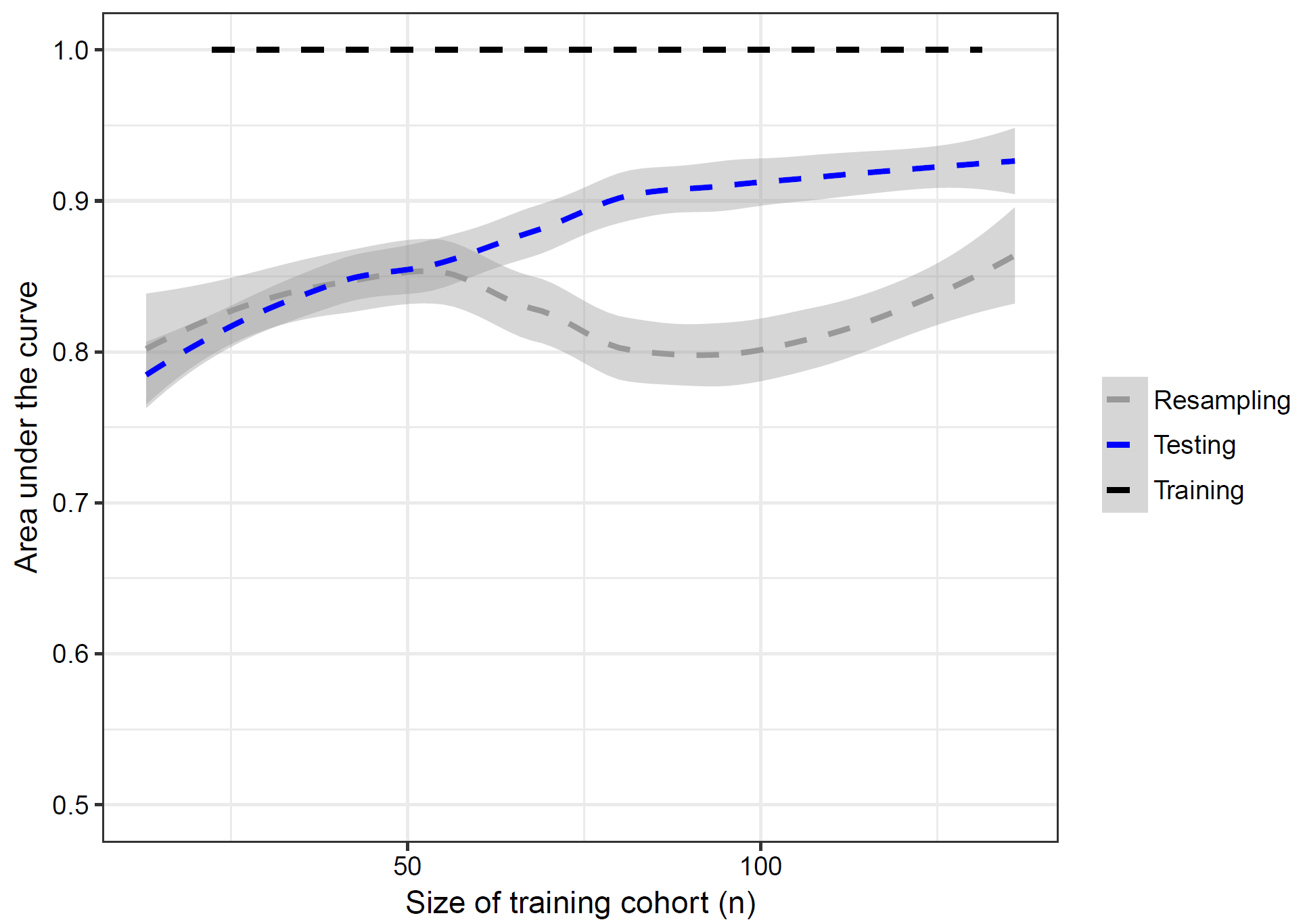


**Figure S6**: Learning curves of the pooled random forest classification illustrate the area under the curve of the training and test set classification performance as a function of the training set size.


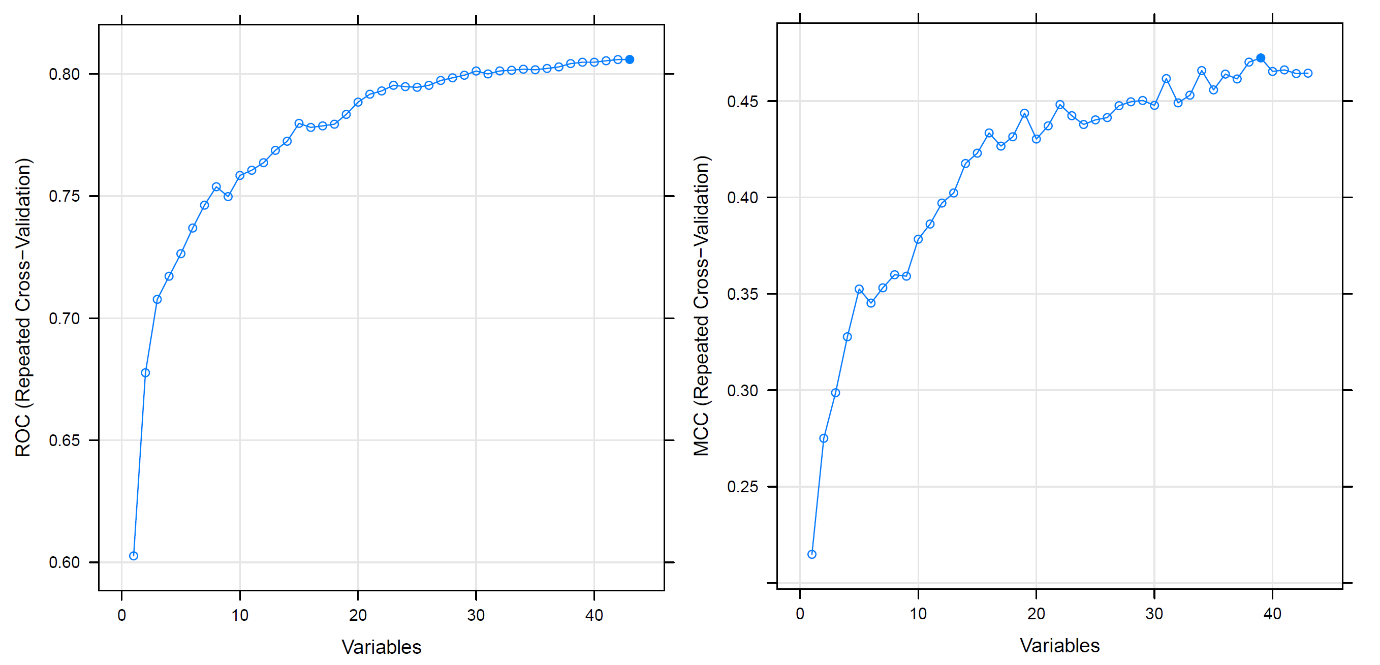


**Figure S7**: Random forest classifier performance measured by are under the receiver operating characteristic curve (AUC; left) and Matthews Correlation Coefficient (MCC; right) displayed as function of the number of independent variables included. The performance plateaus at approximately n=20 features.


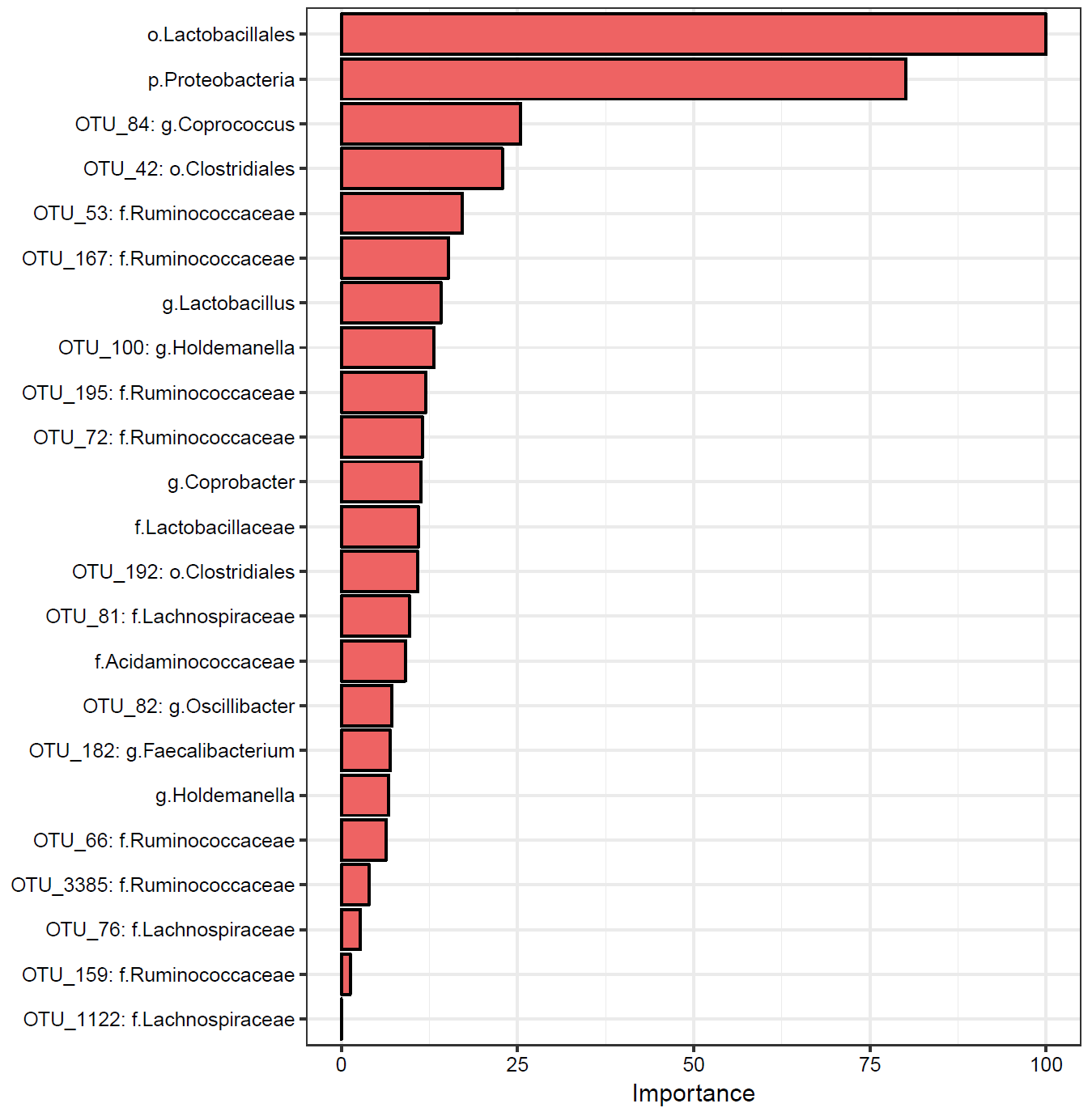


**Figure S8**: Important taxa identified by Boruta algorithm for pooled random forest classification


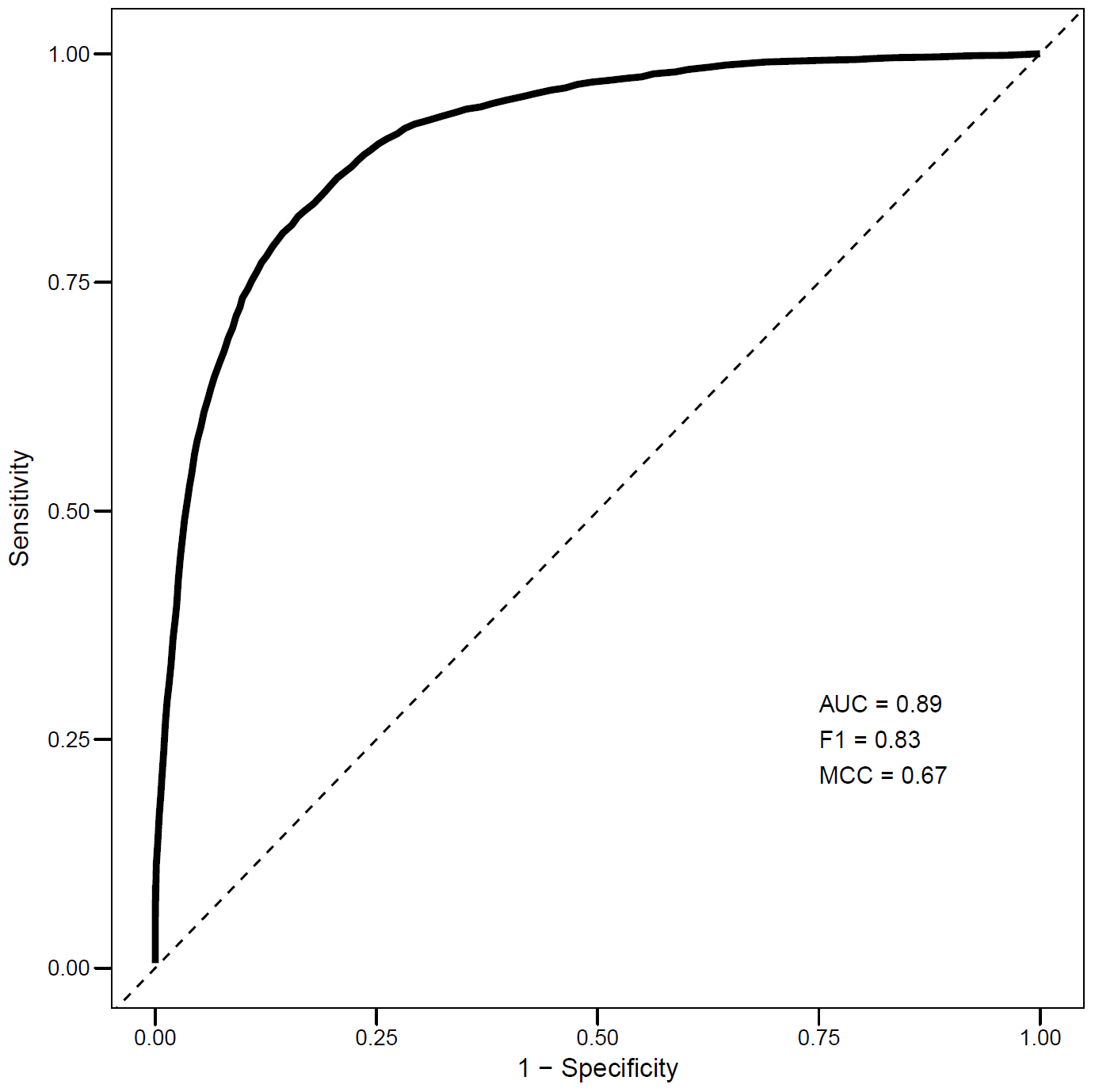


**Figure S9**: Area under the receiver operating characteristic curve for random forest classifier performance with reduced variable set size (n=23). The 0.632 bootstrap estimator was used to estimate the generalization error.

**SUPPLEMENTARY REFERENCES**

[S1] Magro F, Gionchetti P, Eliakim R, et al. Third European Evidence-based Consensus on Diagnosis and Management of Ulcerative Colitis. Part 1: Definitions, Diagnosis, Extra-intestinal Manifestations, Pregnancy, Cancer Surveillance, Surgery, and Ileo-anal Pouch Disorders. Journal of Crohn's and Colitis. 2017;11(6):649-70.

[S2] Wang J, Thingholm LB, Skiecevičienė J, Rausch P, Kummen M, Hov JR, et al. Genome-wide association analysis identifies variation in vitamin D receptor and other host factors influencing the gut microbiota. Nat Genet 2016;48:1396-1406.

[S3 ] Joshi NA, Fass JN. Sickle: A sliding-window, adaptive, quality-based trimming tool for FastQ files (Version 1.33) [Software].  Available at https://github.com/najoshi/sickle.; 2011.

[S4] Edgar RC, Haas BJ, Clemente JC, Quince C, Knight R. UCHIME improves sensitivity and speed of chimera detection. Bioinformatics 2011;27:2194-2200.

[S5] Edgar R. SINTAX: a simple non-Bayesian taxonomy classifier for 16S and ITS sequences. bioRxiv 2016.

[S6] Oksanen J, Blanchet FG, Friendly M, Kindt R, Legendre P, McGlinn D, et al. vegan: Community Ecology Package. R package version 2.4–3.  https://CRAN.R-project.org/package=vegan. 2017

[S7]: Legendre P and Anderson MJ. Distance-based redundancy analysis: Testing multispecies responses in mulfactorial ecological experiments. Ecological Monographs 1999;69:1-24.

[S8] Chen H. 2018; VennDiagram: Generate High-Resolution Venn and Euler Plots. R package version 1.6.20. Available from: https://CRAN.R-project.org/package=VennDiagram

[S9] Shetty A, Hugenholz F, Lathi L, Smidt H, de Vos WM. Intestinal microbiome landscaping: insight in community assemblage and implications for microbial modulation strategies. FEMS Microbiology Reviews 2017; 41, 182-199.

[S10] Kummen M, Holm K, Anmarkrud JA, et al. The gut microbial profile in patients with primary sclerosing cholangitis is distinct from patients with ulcerative colitis without biliary disease and healthy controls. Gut. 2017;66(4):611-9. Epub 2016/02/17.

[S11] Sabino J, Vieira-Silva S, Machiels K, et al. Primary sclerosing cholangitis is characterised by intestinal dysbiosis independent from IBD. Gut. 2016;65(10):1681-9. Epub 2016/05/20.

[S12] Jackman S. pscl: Classes and Methods for R Developed in the Political Science Computational Laboratory. United States Studies Centre, University of Sydney. Sydney, New South Wales, Australia. R package version 1.5.2. URL  https://github.com/atahk/pscl/. 2017 [cited; Available from:

[S13] Venables WN, Ripley BD. Modern Applied Statistics with S. Fourth Edition.  Springer, New York. ISBN  0-387-95457-0 (<http://www.stats.ox.ac.uk/pub/MASS4)>.

[S14] Robinson, M.D., and A. Oshlack. 2010. A scaling normalization method for differential expression analysis of RNA-seq data. Genome Biology 11 (3): R25–R25.

[S15] Kuczynski, J., C.L. Lauber, et al. 2011. Experimental and analytical tools for studying the human microbiome. Nature Reviews Genetics 13 (1): 47–58.

[S16] Robinson, M.D., and G.K. Smyth. 2007. Moderated statistical tests for assessing differences in tag abundance. Bioinformatics 23 (21): 2881–2887.

[S17] Wright MN , Ziegler A. ranger: A Fast Implementation of Random Forests for High Dimensional Data in C++ and R. J Stat Softw 2017;77:1-17.

[S18] Efron B. Estimating the Error Rate of a Prediction Rule: Improvement on Cross-Validation. Journal of the American Statistical Association 1983;78:316-331.

[S19] Matthews BW. Comparison of the predicted and observed secondary structure of T4 phage lysozyme. Biochimica et Biophysica Acta (BBA) - Protein Structure 1975;405:442-451.

[S20] Kuhn M and Johnson K. Applied Predictive Modeling. Springer, New York, 2013.

[S21] Deng H. Guided Random Forest in the RRF Package. 2013;arXiv:1306.0237.

[S22] Meyer D, Dimitriadou E, Hornik K, Weingessel A, Leisch F. e1071: Misc Functions of the Department of Statistics, Probability Theory Group, TU Wien. 2017; Available from: https://cran.r-project.org/web/packages/e1071/index.html

[S23] Chen T, He T. xgboost: eXtreme Gradient Boosting. 2018;R package version 0.6.4.1; Available from: https://cran.r-project.org/web/packages/xgboost/vignettes/xgboost.pdf

[S24] Kursa MB, Rudnicki WR. Feature Selection with the Boruta Package. Journal of Statistical Software 2010;36:1-13.

[S25] Tang R, Wei Y, Li Y, et al. Gut microbial profile is altered in primary biliary cholangitis and partially restored after UDCA therapy. Gut. 2017.
